# The Graphene Structure’s Effects on the Current-Voltage and Photovoltaic Characteristics of Directly Synthesized Graphene/n-Si(100) Diodes

**DOI:** 10.3390/nano12101640

**Published:** 2022-05-11

**Authors:** Šarūnas Jankauskas, Rimantas Gudaitis, Andrius Vasiliauskas, Asta Guobienė, Šarūnas Meškinis

**Affiliations:** Institute of Materials Science, Kaunas University of Technology, K. Baršausko St. 59, LT-51423 Kaunas, Lithuania; sarunas.jankauskas@ktu.lt (Š.J.); rimantas.gudaitis@ktu.lt (R.G.); andrius.vasiliauskas@ktu.lt (A.V.); asta.guobiene@ktu.lt (A.G.)

**Keywords:** graphene, MW-PECVD, photovoltaics

## Abstract

Graphene was synthesized directly on Si(100) substrates by microwave plasma-enhanced chemical vapor deposition (MW-PECVD). The effects of the graphene structure on the electrical and photovoltaic properties of graphene/n-Si(100) were studied. The samples were investigated using Raman spectroscopy, atomic force microscopy, and by measuring current–voltage (I-V) graphs. The temperature of the hydrogen plasma annealing prior to graphene synthesis was an essential parameter regarding the graphene/Si contact I-V characteristics and photovoltaic parameters. Graphene n-type self-doping was found to occur due to the native SiO_2_ interlayer at the graphene/Si junction. It was the prevalent cause of the significant decrease in the reverse current and short-circuit current. No photovoltaic effect dependence on the graphene roughness and work function could be observed.

## 1. Introduction

Graphene, the carbon 2D material, was discovered recently [1]. Notably, graphene’s exciting abilities, such as 97.7% optical transparency [2], the high charge carrier mobility of 200,000 cm^2^ V ^−1^ s^−1^ [3], and Young’s modulus of 1 TPa [4], make it a perfect candidate material for optoelectronic device fabrication [5,6]. One of the prominent features of graphene is that it can be used instead of metal to form a Schottky junction with semiconductors, e.g., silicon [7]. This enables the use of a graphene/silicon (Gr/Si) contact as a base for solar cell production (see reviews [7,8,9,10,11,12,13,14,15]). Today, the highest power conversion efficiency (PCE) reported for Gr/Si solar cells is 16.61% [16]. That is a result of the 9-year development of the Gr/Si contact devices, from the 1.5% conversion efficiency reported for the first graphene/silicon Schottky contact-based solar cell [17]. It means that solar cells based on graphene can be very promising and achieve high PCE. Notably, according to the simulations, it was suggested that the conversion efficiency of the graphene/Si solar cell could potentially reach values higher than the conversion efficiency of the best fabricated solar cells (see [18] and [13], respectively). High-efficiency graphene/silicon solar cells were fabricated by combining silicon surface passivation with ultra-thin dielectric interlayers, graphene doping, and light management techniques such as Si substrate micro/nanotexturing and, especially, antireflective films [7,8,9,10,11,12,13,14,15,19].

Further increase of the graphene/Si solar cell conversion efficiency requires optimization of all the functional parts of the solar cell. In most studies, graphene is synthesized by chemical vapor deposition (CVD) on copper foil and then transferred to the silicon substrate [7,8,9,10,11,12,13,14,15]. The transfer is a prolonged process during which graphene is contaminated by different adsorbates [20], and cracks can be induced in the transferred graphene [21]. It can deteriorate the graphene/silicon junction device’s properties resulting in complicated interface and solar cell property control [22,23]. It was reported that the use of the few-layer graphene significantly increased graphene/Si solar cell efficiency up to 3–4 times [24,25]. However, on copper foil, usually, single-layer graphene is synthesized by chemical vapor deposition [26]. Therefore, few-layer graphene for graphene/Si solar cells is fabricated by the even more complex one-by-one transfer method [22,25,27,28].

The abovementioned problems can be solved using graphene directly grown on silicon by plasma-enhanced chemical vapor deposition [29], although only a few studies have been reported [30,31,32,33,34,35,36,37]. The polycrystalline nature of PECVD graphene increases its defect density compared to that of the transferred graphene grown by CVD on the copper foil [20]. Vertical graphene is more widely used [30,31,34,37] as opposed to its planar counterpart in terms of direct growth on Si. However, it poses additional light absorption issues (see [30,31] and [26,38]). These effects should be considered while optimizing directly synthesized graphene-based solar cells. It is noteworthy that the high defect density transferred graphene interlayer can improve the graphene/Si solar cell’s conversion efficiency compared to the very low defect density transferred graphene monolayer/Si solar cell without the interlayer [39]. Despite increased sheet resistance and defect density, the graphene nanowall/Si photovoltaic device’s open-circuit voltage increased with Schottky barrier height [37]. The photovoltaic conversion efficiency of the transferred GNWs/n-Si solar cell reached up to 4.99% [40]. It was comparable to or even better than the efficiency of the transferred CVD graphene/n-Si solar cells fabricated without the passivating interlayer, surface texturing, doping, or antireflective film. That result was achieved despite much higher defect density in graphene nanowalls compared to the planar graphene grown by CVD on copper foil (4.98% in [25], 0.86% in [41], 3.5% in [13], 1.9–3% in [42]). The graphene nanowall/n-Si solar cell open-circuit voltage increased with graphene layer number despite increased defect density [30]. There are no studies regarding the graphene layer number and defect density influence on photovoltaic properties of the directly synthesized planar graphene and silicon solar cells. Meanwhile, graphene nanowall and transferred CVD graphene cases have their specific peculiarities. Particularly, multilayer graphene fabricated using layer-by-layer transfer results in different orientations of the carbon hexagons in different layers. That may be a reason for the contradictory results concerning optimal graphene layer number and the maximum conversion efficiency achieved [22,24,27,28,38]. Notably, the optimal graphene layer number in different studies varied from two to four (2 in [38], 2–3 in [27], 3 in [28], 4 in [24]). A summarized benchmark showing PCE values and PCE enhancement techniques of the CVD-synthesized graphene/Si solar cells investigated by different research groups can be seen in Table S1.

Therefore, the present study investigates the effects of the directly synthesized graphene structure on current–voltage characteristics and photovoltaic properties of the graphene/n-Si photovoltaic devices. Various synthesis conditions were used to grow graphene samples of different structures and surface morphologies. Only a small influence or no influence of the graphene thickness, defect density, surface morphology, and work function was found. The impact of substrate-induced self-doping and silicon surface pretreatment on the graphene/n-Si device’s current-voltage and photovoltaics characteristics was revealed.

## 2. Materials and Methods

Samples were produced using a microwave PECVD system Cyrannus (Innovative Plasma Systems (Iplas) GmbH, Troisdorf, Germany). Monocrystalline, double-side polished, n-type Si(100) (Sil’tronix Silicon Technologies, Archamps, France), with a resistivity of 1–10 Ω·cm, was used as a substrate. A precursor gas mixture of hydrogen and methane was used for graphene synthesis. Before the growth of graphene, hydrogen plasma was ignited, and methane gas was only introduced when the target temperature was reached. In some cases, the silicon substrates were plasma pre-annealed at higher temperatures than the temperature of the subsequent graphene synthesis. A special enclosure was used to protect from direct plasma that results in high etching rates of the sample (Figure S1). Synthesis parameters for each sample can be seen in Table 1. Samples were grouped into three categories (A–C) based on the Si(100) substrate plasma pre-annealing temperature (700–900 °C). The sample size was 1 × 1 cm.

**Figure 1:**
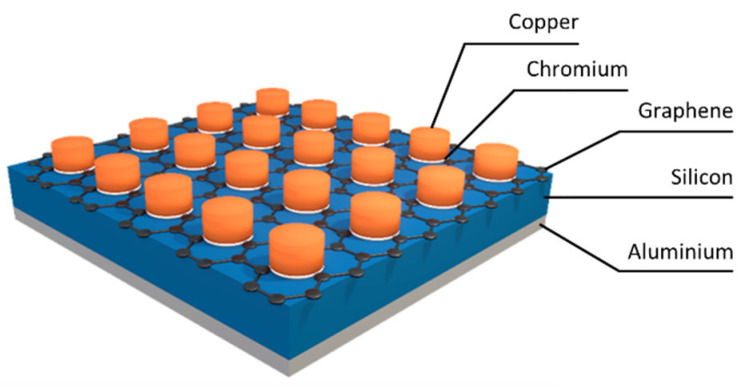
Schematic diagram of the graphene/n-Si(100) fabricated diodes.

After graphene synthesis was carried out, diode fabrication began with Al back contact formation (on the uncoated Si side) using e-beam technology. The DMF + acetone boiling and RCA 1 (1:1:5 solution of NH_4_OH + H_2_O_2_ + H_2_O), impurity removal (1:50 solution of HF + H_2_O), and RCA 2 (1:1:6 solution of HCl + H_2_O_2_ + H_2_O) treatments [43] were done prior to the deposition of the Al layer. Cr/Cu electrodes were deposited on the graphene through a mask with 500 µm circular holes. The thicknesses of the Cr interlayer and Cu layer were 20 and 200 nm, respectively. The schematic diagram is shown in Figure 1. The structure of the device is more similar to the real silicon solar cells than the usually used graphene/Si solar cells, with an active device part consisting of graphene on silicon in a hole opened in the silicon dioxide and metal electrodes on the graphene-coated SiO_2_ [7,8,9,10,11,12,13,14,15]. It should be noted that graphene/Si solar cells of structure similar to ours were fabricated and investigated in [23,44,45,46]. The geometry of the device’s metal electrodes was not optimized.

Thickness and defect characterization was carried out via Raman scattering spectroscopy using a Raman spectrometer, InVia (Renishaw, Wotton-under-Edge, UK). The measurement was done just after the graphene synthesis and before the graphene/Si(100) diode fabrication. We acquired Raman spectra at several different places on each sample, considering possible differences in the graphene structure across the specimen. The beam power was set to 1.5 mW, and the excitation wavelength was 532 nm. Several peaks were analyzed for in-depth characterization and defect estimation (D, G, and 2D). The G peak was separated into two components, with the actual G peak being at 1600 cm^−1^ and the D^’^ peak (which was not analyzed) being at 1620 cm^−1^. The Lorentzian function was used for the best peak fit, considering Merlen et al. [47] who made observations to determine peak intensities, positions, and full width at half maximums (FWHM). The well-known I_D_/I_G_ ratio was used to reveal the defectiveness of our produced samples [48]. In contrast, the I_2D_/I_G_ ratio contributed to the graphene thickness evaluation [49] (smaller ratios correspond to more graphene layers). The positions and FWHM of the G and 2D peaks were analyzed to get information on graphene crystallite size, strain, and doping [50,51,52].

Atomic force microscopy (AFM) was employed to detect any structural peculiarities of the graphene surface. The surface morphology was investigated at room temperature and ambient air conditions using a NanoWizard III atomic force microscope (JPK Instruments, Bruker Nano GmbH, Berlin, Germany). The measurements were done in tapping mode. The silicon probes (CS Instrument, Harrislee, Germany) with a thin layer (25 ± 5 nm) of Pt/Ir coating on both re-ex and tip sides of the probes were used. The probe parameters were as follows: spring constant 2.7 N/m; 60 kHz frequency; 30 nm tip ROC; pyramidal shape. Images of 2 µm × 2 µm size were acquired from the measured data using JPKSPM Data Processing software (version spm-4.3.13, JPK Instruments, Berlin, Germany). Kelvin probe measurements were carried out using the same instrumental setup to evaluate graphene work function.

The current-voltage (I-V) characteristics were measured using a Keithley 6487 picoampere meter/voltage source. The measurements were done at several points on the sample to evaluate the possible dispersion of the characteristics. Characteristics were investigated in three different regimes to study the photovoltaic properties of the fabricated devices. These were dark mode (sample was not illuminated), UV mode (when the sample was illuminated by 406 nm wavelength light-emitting diode (LED)), and IR mode (when the sample was illuminated by 800 nm wavelength light-emitting diode). In all instances, the voltage range was from −2 to +2 V. To ensure the same optical power (5.2 mW) between different measurement modes, currents supplied to the LEDs were selected accordingly. The measurements were done at several different places on the samples to evaluate the dispersion of the results. Diode behavior was studied by examining the I-V characteristic parameters in the dark (reverse current at 0.3 V (I_R_(0.3 V)), forward current vs. reverse current at ±0.1V (I_R_(0.1 V)/I_F_(0.1 V)), forward current vs. reverse current at ±0.3 V (I_R_(0.3 V)/I_F_(0.3 V))). The photovoltaic parameters (short-circuit current (I_SC_) and open-circuit voltage (U_OC_)) were derived from current–voltage characteristics measured under illumination. The I-V characteristic’s dependence on temperature was measured using a similar setup to the photovoltaic parameter measurements. The same Keithley 6487 picoampere meter/voltage source was employed, with thermal operational conditions being changed by a custom-made Peltier element configuration. The temperature varied from −20 to 40 °C. Each measurement was made after the temperature value had settled down.

## 3. Results

### 3.1. Raman Spectra, Current–Voltage Characteristics of Produced Samples and Their AFM Micrographs

**Figure 2:**
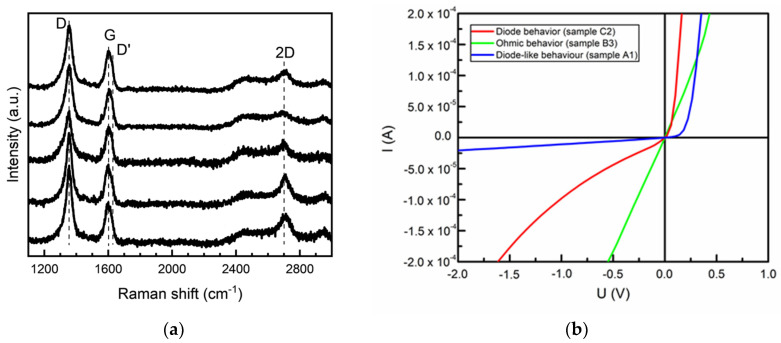
Typical Raman scattering spectra (**a**) and typical I−V characteristics (**b**) of directly synthesized graphene/Si(100) devices. The I−V characteristics of the device produced from the C2 sample exhibited diode behavior (red), ohmic contact was seen for the device produced from a sample B3 (green), and the A1 sample had diode-like I-V features (blue).

The Raman fingerprints of the synthesized samples were investigated, and graphene-related peaks were confirmed (Figure 2a) [53]. The 2D peak was observed at ~2700 cm^−1^. The G peak of our samples lay at ~1600 cm^−1^. All synthesized samples had a prominent defect-related D peak at ~1350 cm^−1^. The D’ band was detected at ~1620 cm^−1^ as a shoulder of the G peak. This is also a significant feature showing the presence of the defects in the graphene sample [47,54]. The defect-related peaks are due to the nanocrystalline nature of the directly synthesized graphene [29,55]. This was also confirmed by the I_D‘_/I_D_ ratio, which was in the 2.62–4.6 range, indicating that the dominant defect source was grain boundaries [54,56]. The further analysis of the selected samples will be discussed in later sections.

The typical current–voltage (I-V) characteristics of the produced photodiodes can be seen in Figure 2b. It is clear that even though directly synthesized graphene/n-Si(100) devices mostly showcase diode behavior (as is expected), exceptions such as ohmic device operation regimes were found.

The graphene AFM images and topography parameters were studied to supplement our Raman spectroscopy findings (Figures S2–S10, Table 2). The I_2D_/I_G_ ratio values indicated the presence of few-layer graphene. The thickness of the one graphene layer was ~0.4 nm [57,58]. Thus, according to the roughness values larger than several nm, non-planar graphene was grown in some samples (Table 2, Figures S2, S5, S7–S9) [27]. Sample roughness ranged from 0.19 to 5.2 nm, indicating different surface morphologies. The work functions calculated from measured contact potential (VCPD) averaged at 4.820–4.826 eV (Table 2) despite different growth conditions. Thus, the work function variation was tiny.

### 3.2. Raman Scattering Spectra Parameters and Synthesized Graphene Thickness, Defect Density, Doping, and Stress

In most cases, graphene Raman scattering spectra were investigated for defect-free or few-defect graphene (no Raman D peak). However, directly synthesized graphene usually contains a significant number of defects [55]. This can affect several Raman D, G, and 2D peak parameters. In addition, graphene layer number, doping, and stress can also significantly impact its Raman spectra [59,60]. Therefore, a more in-depth investigation of several aforementioned peak parameters was carried out.

**Figure 3:**
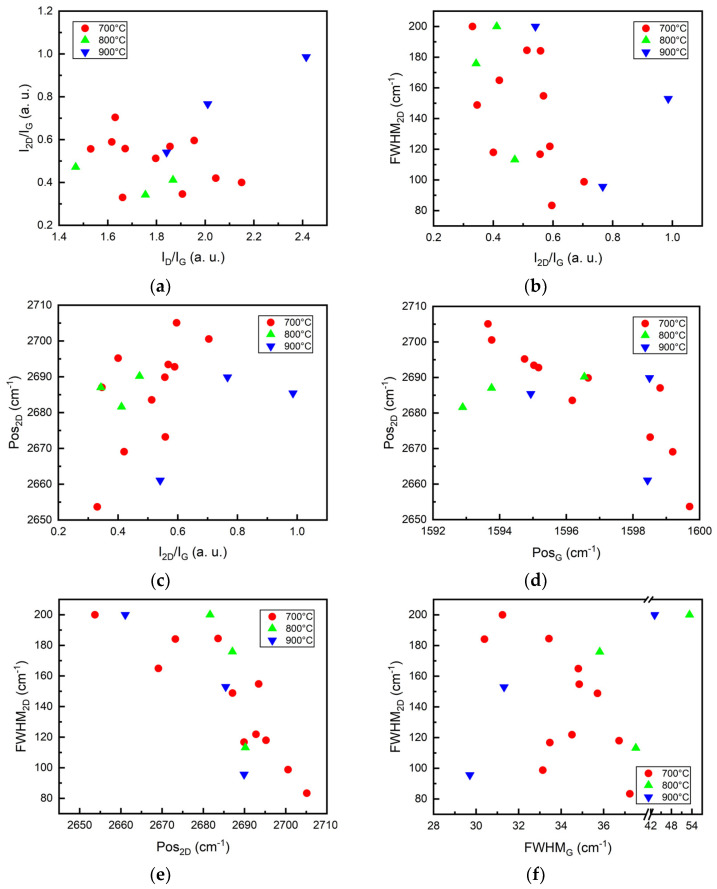
Relation between different Raman scattering spectra parameters: (**a**) I_2D_/I_G_ vs. I_D_/I_G_; (**b**) FWHM_2D_ vs. I_2D_/I_G_; (**c**) Pos_2D_ vs. I_2D_/I_G_; (**d**) Pos_2D_ vs. Pos_G_; (**e**) FWHM_2D_ vs. Pos_2D_; (**f**) FWHM_2D_ vs. FWHM_G_. Samples were grouped according to the temperature of Si(100) substrate hydrogen plasma annealing before graphene growth: 700 °C (red), 800 °C (green), 900 °C (blue).

Remarkably, the decreased intensity of the 2D peak is commonly observed in defected graphene, including that grown by direct synthesis [61,62]. However, no decrease in the I_2D_/I_G_ ratio with I_D_/I_G_ ratio was found (Figure 3a). Thus, the I_2D_/I_G_ ratio can still be used to evaluate graphene layer number, as suggested in [49].

The width of the 2D peak increases, and the peak position upshifts with increased graphene layer number (decreased I_2D_/I_G_ ratio) [49]. One can see only a weak tendency of the FWHM_2D_ decrease with the I_2D_/I_G_ ratio increase in Figure 3b. Very different FWHM_2D_ values can be found for graphene samples of the same thickness. Thus, FWHM_2D_ depends on some other factors. The Pos_2D_, in our case, was upshifted with the I_2D_/I_G_ ratio (Figure 3c). In contrast, the 2D peak should downshift with decreased layer numbers [49]. Thus, no Pos_2D_ dependence on graphene layer number was revealed. Therefore, doping or strain effects can be the origin of the significant differences between the 2D peak position and FWHM_2D_ of the different graphene samples [60,63,64,65].

FWHM_G_ is related to the I_D_/I_G_ ratio of defective graphene [66]. However, no clear FWHM_G_ dependence on I_D_/I_G_ ratio was found in our case (Figure S11a).

The FWHM_G_ decreases with increased crystallite size [66,67,68] and graphene doping [63,69,70,71]. The latter case is accompanied by a Pos_G_ shift to the higher wavenumbers [70]. At the same time, a slight narrowing of the G peak with Pos_G_ upshift was seen (Figure S11b). Thus, the doping effects on G peak narrowing can be supposed. However, the influence of the crystallite size changes cannot be rejected.

The Pos_2D_ vs. Pos_G_ plot can be used to separate compressive and tensile stress and p-type and n-type doping effects [60,65,69,72]. The downshift of the Pos_2D_ with the upshift of the Pos_G_ was found (Figure 3d). It is a signature of n-type doping [60,73]. The FWHM_2D_ decreased with an upshift of the Pos_2D_ (Figure 3e). This is similar to the case in [73], where such behavior was reported for n-type doped graphene. Thus, according to Figure 3d,e, the synthesized graphene samples are n-type self-doped. The 2D peak is downshifted and broadened with increased n-type dopant density [73].

It should be mentioned that the presence of the strain in graphene results in the FWHM_2D_ linear increase with FWHM_G_ [63,74]. Meanwhile, in Figure 3d, FWHM_2D_ increase with FWHM_G_ can be seen only for three samples that were grown on Si(100) pre-annealed at 900 °C. For the samples synthesized on the silicon pre-annealed at 700 °C temperature, the tendency of the FWHM_2D_ to decrease with increased FWHM_G_ was found (Figure 3f). This supports the assumption of n-type self-doping of the studied graphene [63,73]. Different sizes of the graphene crystallites can explain the significantly different FWHM_2D_ values seen for samples with nearly the same FWHM_G_ values [50]. Thus, one can suppose that the charge transfer from the Si(100) substrate to the graphene occurs during the graphene growth, resulting in the n-type self-doping of the graphene. This explanation was provided in [75], taking into account [76,77,78].

### 3.3. Current–Voltage Characteristics’ Relation with Raman Parameters of Fabricated Graphene/Si Devices

The relations between the current–voltage (I-V) characteristics of the graphene/Si(100) heterojunctions and graphene structure were studied. The initial surface preparation significantly influences the Schottky and ohmic contact I-V characteristics [23,79,80,81]. Therefore, we separately analyzed graphene samples synthesized on the silicon substrate, with hydrogen plasma-treated at different temperatures, to discern the graphene structure and the graphene/Si interface effects. Hydrogen plasma’s silicon surface treatment was widely studied and used for amorphous hydrogenated silicon and monocrystalline silicon heterojunctions. However, their mechanisms are far from the final description due to the complexity of the competing effects. That is an increase of silicon surface roughness [82], silicon etching [83] and etching rate dependence on temperature [84,85], Si surface amorphization [82], defect generation [86,87], and different silicon hydrides’ creation [86].

**Figure 4:**
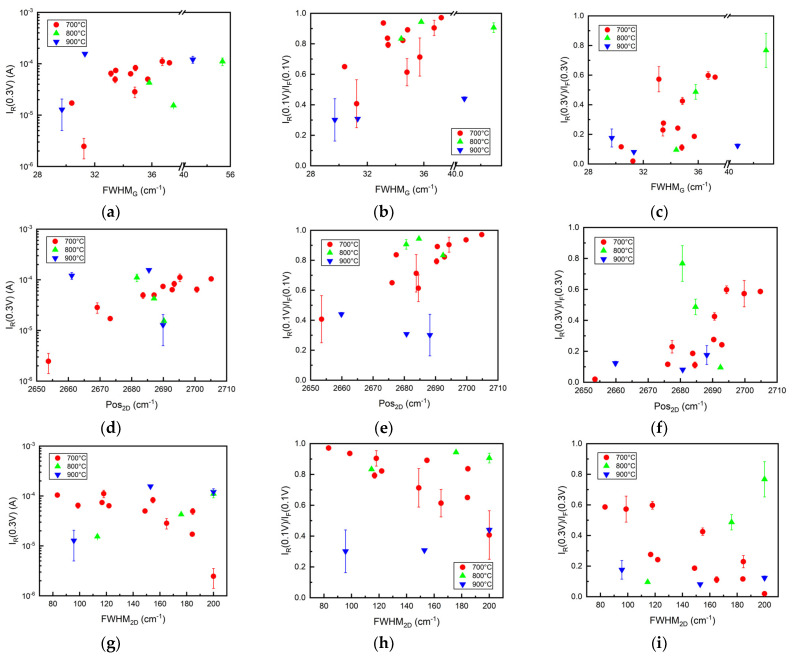
I−V characteristic parameters (I_R_(0.3 V), I_R_(0.1 V)/I_F_(0.1 V), I_R_(0.3 V)/I_F_(0.3 V)) in relation with: (**a**–**c**) FWHM_G_; (**d**–**f**) Pos_2D_; and (**g**–**i**) FWHM_2D_. Samples were grouped according to the temperature of Si(100) substrate hydrogen plasma annealing before graphene growth: 700 °C (red), 800 °C (green), 900 °C (blue).

No clear dependence of the different I-V characteristic parameters (I_R_(0.3 V), I_R_(0.1 V)/I_F_(0.1 V), I_R_(0.3 V)/I_F_(0.3 V)) on the main Raman peak ratios was found (Figure S12). However, the G peak broadening influences the I-V characteristics’ shape (Figure 4a–c). As FWHM_G_ approaches higher values, indicating lowered self-doping level and, possibly, graphene crystallite size decrease [50], the reverse current rises. The reverse and forward current ratios approach 1, implying the ohmic behavior of the junction (Figure 4b,c). Differences between sample groups are not that noticeable, apart from samples annealed at 900 °C, which resulted in a smaller current ratio. We noticed a general increase of reverse current and reverse/forward current ratios, with 2D peak blueshift (ranging from 2653 to 2705 cm^−1^), when the Si(100) substrate was hydrogen plasma pre-annealed at 700 °C (Figure 4d–f). Considering the analysis provided in the Section 3.2, the reverse current and I_R_/I_F_ ratios decrease with increased n-type self-doping levels [59]. When looking at other sample groups, results were inconclusive, although samples annealed at 800 °C showcase a much different trend in current ratios at 0.1 V, with the current ratio dropping when Pos_2D_ increases. When analyzing FWHM_2D_ dependence on reverse current and I_R_(0.3 V)/I_F_(0.3 V) ratio, it is seen that values of the reverse current and I_R_(0.3 V)/I_F_(0.3 V) of the samples grown after annealing in 700 °C gradually decrease when FWHM_2D_ decreases (Figure 4g–i). Thus, it supports the premise that an increased n-type self-doping level decreases the reverse current and I_R_/I_F_ ratio [59].

Inclusions of the non-planar graphene, such as wrinkles, can significantly influence charge transport properties [88,89]. However, results are somewhat inconsistent when analyzing I-V parameters and their relation to roughness (Figure S13). The general tendency of I_R_(0.3 V) decrease (I_R_(0.3V)/I_F_(0.3 V) increase) with increasing surface roughness can be observed, although the plot’s strange “branching out” is seen. Due to this, it is impossible to conclude whether this magnitude of roughness impacts device performance.

### 3.4. Photovoltaic Characteristics of Fabricated Graphene/Si Devices and Their Relation to the Raman Parameters of the Produced Graphene

**Figure 5:**
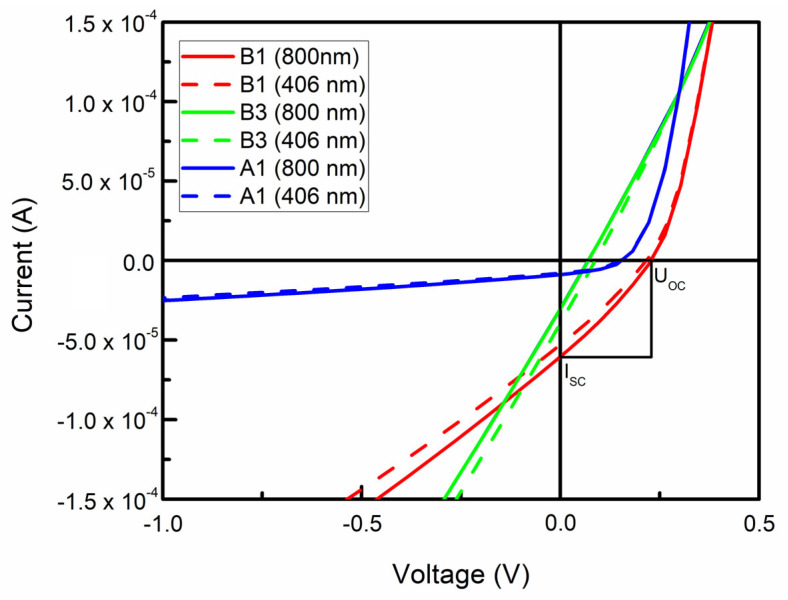
Typical I−V characteristics of directly synthesized graphene/Si heterojunctions measured under the illumination by 800 nm wavelength (solid) and 406 nm wavelength (dashed) LEDs.

Typical I-V curves of produced photovoltaic devices under illumination can be seen in Figure 4. Differences between different illumination regimes are minimal, with 800 nm excitations, in most cases, contributing to a more significant photovoltaic effect, as presumed (Figure 5, Figure S14). The shape of the I-V characteristics in the fourth quadrant is typical for graphene/n-Si solar cells grown without the intentional graphene doping and intentionally deposited ultra-thin dielectric interlayers [24,32,44,90,91]. No S-shaped I-V characteristics reported for some graphene/Si solar cells [92,93,94] were found. 

**Figure 6:**
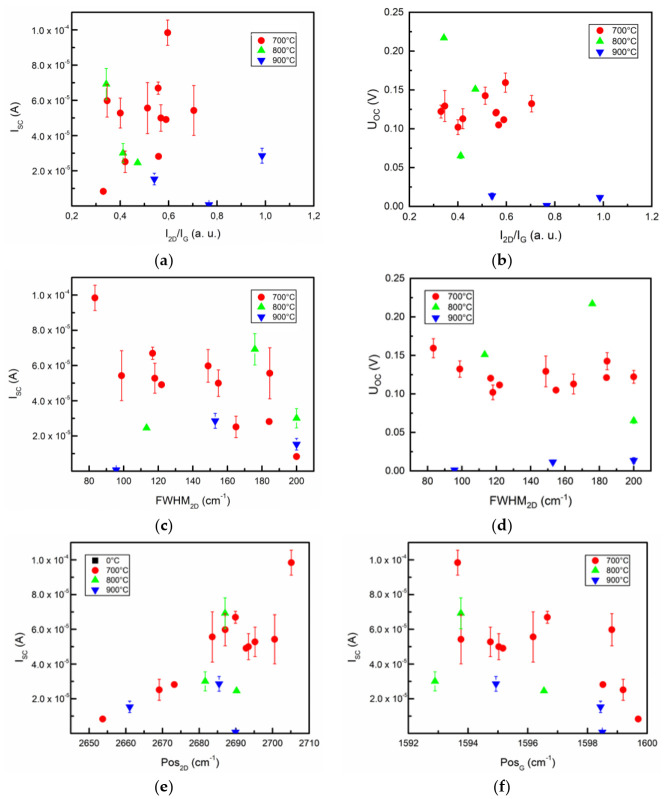
Structural effects on graphene/Si(100) diode’s photovoltaic parameters at 800 nm illumination: (**a**) I_SC_ vs. I_2D_/I_G_ plot; (**b**) U_OC_ and I_2D_/I_G_ relation; (**c**) I_SC_ relation with FWHM_2D_; (**d**) U_OC_ vs. FWHM_2D_ plot; (**e**) I_SC_ with respect to Raman 2D peak position; and (**f**) I_SC_ and Pos_G_ correlation. Samples were grouped according to the temperature of Si(100) substrate hydrogen plasma annealing before graphene growth: 700 °C (red), 800 °C (green), 900 °C (blue).

To analyze the effects of the graphene structure on photovoltaic properties of the graphene/Si(100) samples, I_SC_ and U_OC_ were investigated concerning Raman parameters (Figure 6). Figure 5a shows an I_SC_ of our fabricated Cu/Cr/Gr/Si/Al device in relation to the I_2D_/I_G_ ratio of synthesized graphene. The same investigation scheme was chosen due to the previously mentioned effects of hydrogen plasma annealing before graphene growth. Devices show little to no correlation between photovoltaic parameters and I_2D_/I_G_. Only samples that were annealed at 700 °C exhibited some increase in I_SC_ and U_OC_ when I_2D_/I_G_ increased (layer number decreases) (Figure 6a,b). The samples annealed at 900 °C distinctly produced the lowest I_SC_ and U_OC_. Thus, the surface pre-treatment conditions are more critical than the graphene layer number regarding the photovoltaic parameters. Considering the changes of the U_OC_ and I_SC_ in the samples grown using 700 °C temperature pre-treatment, the graphene layer number effects can be explained by changes in the reflectance, optical transmittance, and graphene work function [27]. In the graphene/Si solar cell, the open-circuit voltage increase was explained by the rise in the Schottky barrier height and work function [24,30]. In our case, no dependence of the graphene/Si solar cell short-circuit current and open-circuit voltage on graphene work function was found. As mentioned earlier in this article, the graphene layer number necessary for maximization of the graphene/Si solar cell photovoltaic characteristics was reported by different authors to be from two to four [24,27,28,38]. In our case, the lowest graphene layer number used, according to the I_2D_/I_G_ ratio analysis, was 1–2 layers. Thus, our results are close to the data reported in [28,38], where no graphene work function influence was revealed. Noteworthily, I_SC_ exhibited a noticeable decrease with increased FWHM_2D_ (Figure 6c) when samples were annealed at 700 °C. Similar results were not reproduced when looking at the U_OC_–FWHM_2D_ relation (Figure 6d), with samples occupying similar values of U_OC_ throughout the whole range of FWHM_2D_. 

We also analyzed the I_SC_ correlation with Pos_2D_ and Pos_G_ (Figure 6e,f). Interestingly enough, almost all analyzed samples followed an increasing I_SC_ trend with a shift of the Pos_2D_ to the higher wavenumbers and Pos_G_ to the lower wavenumbers. It means that the graphene n-type self-doping could be the predominant phenomenon, affecting photovoltaic properties [75] (Table S2). Thus, the graphene n-type self-doping results in decreased short-circuit current. That is in accordance with numerous studies because graphene p-type doping is used to increase graphene/n-Si solar cell efficiency by raising the graphene/Si contact potential barrier height [10]. The same distribution could not be recorded for U_OC_ due to very dispersive data (Figure S15).

The photovoltaic properties’ relation with I_D_/I_G_ and FWHM_G_ plots was employed to examine changes in electric properties due to defects or grain size effects (Figure S16). Relatively high dispersion can be seen when analyzing short circuit current deviation due to defects (in terms of I_D_/_G_) (Figure S16a,b), and data distribution gives no concrete answer. When observing defect influence on open-circuit voltage, higher U_OC_ values did not correlate to the aforementioned parameters (Figure S16). It should be mentioned that, in [31,39,40], graphene/Si solar cell conversion efficiency was improved by inserting a highly defective graphene interlayer. While in [30], the lowest U_OC_ and I_SC_ were found for directly synthesized graphene/Si solar cells fabricated using graphene with the lowest defect density. It is also hard to stress any presence of photovoltaic parameter variation due to grain size [50] after analyzing G band broadening (Figure S16c,d). Data points are too dispersive to conclude. When considering sample topography and its significance on photovoltaic parameters, it is essential to note that only a small I_SC_ reduction can be seen due to the increase in roughness (Figure S17). The most notable case is samples grown on the Si(100) annealed at 800 °C. In the case of the 700 °C annealing, no correlation can be observed due to predominant roughness effects. U_OC_ and RMS roughness relation indicate relatively high dispersion, thus omitting roughness as a detrimental parameter of open-circuit voltage. It should be mentioned that transferred CVD graphene/Si solar cell efficiency can be improved by inserting a graphene nanowall interlayer [38]. At the same time, the transferred graphene nanowalls and n-Si solar cell efficiency were comparable to the efficiency of the transferred CVD graphene/n-Si solar cells [40].

### 3.5. I-V and Photovoltaic Parameter Relation

**Figure 7:**
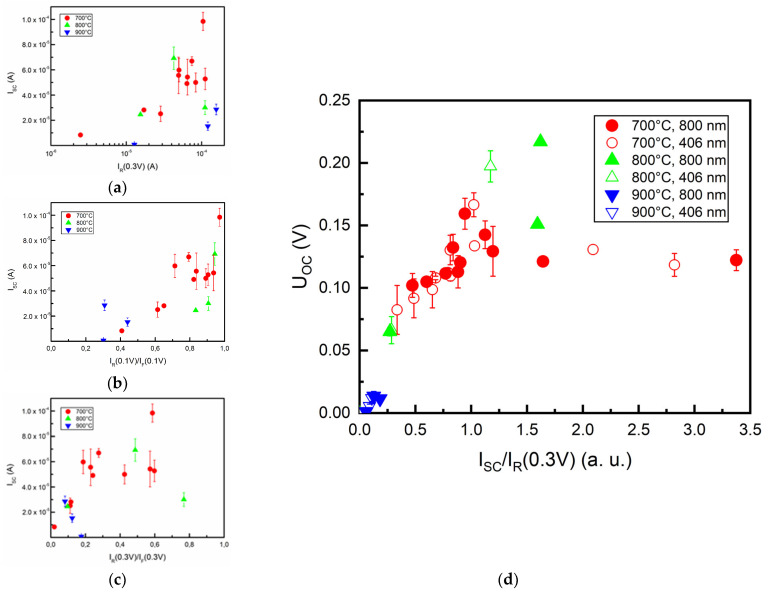
Diode I−V and photovoltaic parameter (at 800 nm illumination) relation: (**a**) I_SC_ vs. I_R_(0.3 V) plot; I_SC_ correlation with diode’s I-V curve shape estimated using reverse and forward current ratio measured at 0.1 V (**b**) and 0.3 V (**c**); U_OC_ vs. I_SC_/I_R_(0.3 V) plot (**d**) (solid and hollow markers indicate device illumination at 800 and 406 nm, respectively).

The photovoltaic parameter’s relation with I_R_(0.3V) was analyzed. An increase in the I_SC_ following a rise in I_R_(0.3V) can be seen at least in two groups of samples (Figure 7a). Curve shape investigation was carried out using reverse and forward current ratios at 0.1 V and 0.3 V, respectively, as the diode nature of samples may impact photovoltaic parameters. In samples that were annealed at 700 °C, an increase in I_SC_ can be seen when I_R_(0.1 V)/I_F_(0.1 V) increases (Figure 7b), with other groups following that tendency dubiously. When I_R_(0.3 V)/I_F_(0.3 V) is taken into account (Figure 7c), the dispersion of data became broad, hence limiting conclusiveness. When analyzing the aforementioned I-V parameters with respect to U_OC_, the results were even more dispersive (Figure S18). The U_OC_ vs. I_SC_/I_R_(0.3 V) plot was employed to show that U_OC_ tends to increase with a short-circuit and reverse current ratio increase, although it branches out when the ratio reaches a value of ~1 (Figure 7d). While annealing temperatures had an impact on I_SC_/I_R_(0.3V), which tends to be minimal (<0.183 V) when annealing was carried out at 900 °C, different illumination regimes show that IR irradiation yields higher U_OC_ and I_SC_. As in many discussed relations, samples that were annealed at 700 °C also had the most significant spread of I_SC_/I_R_(0.3 V), with values situated in a range of 0.336–3.375. U_OC_ in samples that had been annealed at 800 °C before graphene growth tended to increase with I_SC_/I_R_(0.3V), although moderate dispersion of values was observed.

## 4. Discussion

The electron transfer from the n-Si(100) to the graphene should result in decreased graphene/Si contact barrier and, hence, increased reverse current [95,96]. In our case, the opposite tendency was found. However, the native oxide layer can be present at the graphene and silicon interface because silicon surface reoxidation after direct graphene synthesis was reported in [62]. Graphene placed on the silicon dioxide can be electron-doped due to the positive silanol groups on the SiO_2_ surface [97,98].

The charge exchange at the graphene/SiO_2_ interface results in a dipole formation, and charge redistribution imposes *n* doping in the graphene [98], although no chemical bonds form at the graphene–SiO_2_ interface [99]. The graphene placed on the amorphous SiO_2_ can also be n-type doped [99].

It should be mentioned that the single-layer graphene Fermi level and work function vary equally [100]. Nevertheless, in the present study, the graphene samples’ work function changed in a very narrow range despite different graphene n-type doping levels found while evaluating Pos_2D_ (Figure 3d).

Graphene work-function shift with doping significantly decreased when the graphene layer number increased [101]. The main decline occurs with changing from single-layer to two-layer graphene [101]. The work function of the 4–5-layer graphene was the same as that of the pristine undoped ultra-thin graphite [101]. The work function of graphene placed on SiO_2_ decrease (increase) with graphene dopant concentration is significantly suppressed by increasing the graphene layer number [102]. This is because of the charge transfer from SiO_2_ to the graphene and subsequent charge redistribution within the graphene [103]. The charge in graphene decays exponentially with distance from the substrate resulting in suppressed changes in the few-layer graphene work function [101]. Numerous defects found in the directly synthesized graphene by Raman scattering spectroscopy (Figure 2a) can also reduce the graphene’s work function shift [104].

The analysis of samples’ I-V characteristics measured at different temperatures revealed the flow of the tunneling and thermionic emission currents (Supplementary Materials S4). At lower measurement temperatures, the tunneling current dominated (Supplementary Materials S4, Figures S19 and S20). For I-V characteristics measured at higher temperatures of 30 and 40 °C, the current is dominated by the thermionic emission at low reverse biases, and at higher voltages, the tunneling current prevailed (Supplementary Materials S4). The tunneling current via ultra-thin dielectric grown on the n-type semiconductor can be decreased by a fixed positive charge induced in the dielectric layer [94]. The graphene Pos_2D_ should downshift and the FWHM_2D_ should increase with an increase in doping and, hence, increased native oxide surface positive charge density. Thus, the reverse current and I_R_/I_F_ ratios decrease with the graphene substrate-induced self-doping seen in Figure 3d–i is in good accordance with this assumption. In such a way, the I_SC_ increase with I_R_/I_F_ ratio and with I_R_ can be explained by the flow of the tunneling photocurrent similarly to the quantum dot and superlattice solar cells where the tunneling effect was used to raise the short-circuit current [105,106]. It should be mentioned that, in the graphene/ultra-thin dielectric/Si solar cells, short-circuit current increases with tunneling current [32]. In addition, graphene/ultra-thin dielectric/Si photodiodes photoresponsivity also increases with increased tunneling current [107,108].

It was revealed that the silicon substrate hydrogen plasma pre-annealing was a very important technological parameter regarding the photovoltaic parameters. An increase in the annealing temperature to 900 °C resulted in suppression of the photovoltaic effect. The AFM study revealed no clear morphology and phase changes due to the silicon surface treatment by hydrogen plasma at both 700 and 900 °C (Table S3). Si(100) surface plasma annealing at 700 °C resulted in no work function changes. However, plasma treatment at 900 °C decreased the substrate surface work function by ~0.05 eV, indicating a silicon surface electronic structure change (Table S3). Thus, in the present study, the effects of initial substrate surface electronic structure on graphene/Si device photovoltaic properties were more significant than differences in the graphene structure.

U_OC_ did not depend on the I_SC_ and increased with I_SC_/I_R_ ratio for ratios up to 1–1.5 (Figure 7). It can be explained by relatively large dark reverse currents found in studied samples [109]. That is because U_OC_, differently from the I_SC_, usually is decreased due to the tunneling [110]. Reduced U_OC_ with increased leakage current was reported for multi-crystalline silicon [111], organic [112,113], and graphene/GaAs [114] solar cells.

## 5. Conclusions

In conclusion, the graphene synthesis conditions, structure, and substrate treatment’s effects on directly synthesized graphene/n-Si(100) photovoltaic devices properties were revealed.

The graphene n-type self-doping due to the charge transfer from the native SiO_2_ interlayer to the graphene was the main reason for the notable reverse current (I_R_) and short-circuit current (I_SC_) decrease. Due to the tunneling photocurrent flow, the U_OC_ increased with a short-circuit current, and the reverse current ratio increased. Significant hydrogen plasma pre-treatment effects on the current-voltage characteristics and photovoltaic parameters were observed, revealing the importance of the graphene/silicon interface.

It was found that the graphene samples’ work functions were nearly the same (4.820–4.826 eV), even though the graphene structure and properties of the photovoltaic devices varied dramatically. No effects of graphene surface morphology and defects on the electrical and photovoltaic characteristics were found. The short-circuit current and open-circuit voltage only slightly increased with graphene layer number.

Thus, directly synthesized graphene/n-Si solar cells can be improved by preventing n-type self-doping and optimizing the graphene/silicon interface, whereas graphene defects, layer number, work function, and morphology are much less critical.

## Figures and Tables

**Table 1:** Synthesis conditions for investigated graphene samples.

Sample No.	Power, kW	H_2_, sccm	CH_4_, sccm	p, mBar	T, °C	t, min	Annealing Temperature, °C
A1	0.7	75	25	10	700	60	700
A2	0.7	75	25	20	700	60	700
A3	0.7	75	35	10	700	60	700
A4	0.7	75	35	20	700	60	700
A5	0.7	75	35	20	700	60	700
A6	0.7	75	35	20	700	60	700
A7	0.7	150	50	22	700	60	700
A8	0.7	75	25	10	700	90	700
A9	0.7	75	35	20	700	90	700
A10	0.7	75	25	10	700	150	700
A11	0.7	75	25	10	700	150	700
B1	0.7	75	35	10	700	60	800
B2	0.7	75	25	10	700	90	800
B3	0.7	75	35	20	800	60	800
C1	0.7	75	35	20	700	60	900
C2	0.7	75	25	10	700	90	900
C3	0.7	150	50	22	900	20	900

**Table 2:** AFM parameters of the directly synthesized graphene samples.

Sample No.	Highest Surface Point, nm	RMS Roughness, nm	Φ, eV	I_2D_/I_G_
A1	~9	2.1	4.82	0.33
A7	1.3	0.295	4.824	0.42
A8	0.9	0.19	4.824	0.35
A11	3.39	0.77	-	0.6
B1	1.8	0.42	4.824	0.34
B2	~15	3.5	-	0.47
B3	~6	1.36	4.824	0.41
C2	22.9	5.2	-	0.77
C3	1.5	0.332	4.826	0.54

## Data Availability

Not applicable.

## Supplementary Materials

The following supporting information can be downloaded at https://www.mdpi.com/article/10.3390/nano12101640/s1: Figure S1: Schematic of an enclosure that was used during the MW-PECVD process to prevent direct plasma effects; Figure S2: AFM image (a), AFM phase image (b), and height profile (c) of A1 sample; Figure S3: AFM image (a), AFM phase image (b), and height profile (c) of A7 sample; Figure S4: AFM image (a), AFM phase image (b), and height profile (c) of A8 sample; Figure S5: AFM image (a), AFM phase image (b), and height profile (c) of A11 sample; Figure S6: AFM image (a), AFM phase image (b), and height profile (c) of B1 sample; Figure S7: AFM image (a), AFM phase image (b), and height profile (c) of B2 sample; Figure S8: AFM image (a), AFM phase image (b), and height profile (c) of B3 sample; Figure S9: AFM image (a), AFM phase image (b), and height profile (c) of C2 sample; Figure S10: AFM image (a), AFM phase image (b), and height profile (c) of C3 sample; Figure S11: FWHMG vs. ID/IG (a) and FWHMG vs. PosG (b) plots; Figure S12: I-V characteristic parameters: (a,d) IR(0.3 V); (b,e) IR(0.1 V)/IF(0.1 V); (c,f) IR(0.3 V)/IF(0.3 V); in relation with (a-c) I2D/IG; (d-f) ID/IG; Figure S13: I-V characteristic parameters: (a) IR(0.3 V); (b) IR(0.3 V)/IF(0.3 V) in relation with surface roughness; Figure S14: ISC vs. ID/IG (a) and UOC vs. ID/IG (b) plots showing a difference between devices measured at 800 nm illumination (solid) and 406 nm illumination (hollow); Figure S15: UOC vs. PosG plot; Figure S16: ISC (a,c) and UOC (b,d) relation with respect to ID/IG (a,b) and FWHM2D (c,d) under 800 nm illumination; Figure S17: ISC (a) and UOC (b) and sample roughness relation under 800 nm illumination; Figure S18: Diode I-V and UOC (at 800 nm illumination) relation; Figure S19: Different charge transport mechanisms estimated from typical fabricated diode I-V graphs under various thermal conditions: (a) Poole–Frenkel mechanism; (b) Image-force-induced charge transport; (c) Thermionic emission; Figure S20: Diode operating regimes in terms of temperature: (a) typical I-V characteristics measured in the dark at different temperatures (253–313 K); (b) The Arrhenius plot; (c) ln(σ/T2) vs. 1000/T plot; Table S1: Summarized benchmark showing PCE values and PCE enhancement techniques of the CVD-synthesized graphene/Si solar cells investigated by different research groups; Table S2: Probable doping and strain effects governing main graphene’s Raman peak (G, 2D) positions and their FWHM; Table S3: Hydrogen plasma pre-treatment effects on Si(100) substrate surface. References [115,116,117,118,119,120,121,122,123,124,125] are cited in the Supplementary Materials.

## References

[1] Novoselov K.S. (2004). Electric Field Effect in Atomically Thin Carbon Films. Science.

[2] Nair R.R., Blake P., Grigorenko A.N., Novoselov K.S., Booth T.J., Stauber T., Peres N.M.R., Geim A.K. (2008). Fine Structure Constant Defines Visual Transparency of Graphene. Science.

[3] Morozov S.V., Novoselov K.S., Katsnelson M.I., Schedin F., Elias D.C., Jaszczak J.A., Geim A.K. (2008). Giant Intrinsic Carrier Mobilities in Graphene and Its Bilayer. Phys. Rev. Lett..

[4] Lee C., Wei X., Kysar J.W., Hone J. (2008). Measurement of the Elastic Properties and Intrinsic Strength of Monolayer Graphene. Science.

[5] Bonaccorso F., Sun Z., Hasan T., Ferrari A.C. (2010). Graphene Photonics and Optoelectronics. Nat. Photonics.

[6] Li X., Tao L., Chen Z., Fang H., Li X., Wang X., Xu J.-B., Zhu H. (2017). Graphene and Related Two-Dimensional Materials: Structure-Property Relationships for Electronics and Optoelectronics. Appl. Phys. Rev..

[7] Huang K., Yu X., Cong J., Yang D. (2018). Progress of Graphene-Silicon Heterojunction Photovoltaic Devices. Adv. Mater. Interf..

[8] Wirth-Lima A.J., Alves-Sousa P.P., Bezerra-Fraga W. (2019). Graphene/Silicon and 2D-MoS_2_/Silicon Solar Cells: A Review. Appl. Phys. A.

[9] Bhopal M.F., Lee D.W., ur Rehman A., Lee S.H. (2017). Past and Future of Graphene/Silicon Heterojunction Solar Cells: A Review. J. Mater. Chem. C.

[10] Kong X., Zhang L., Liu B., Gao H., Zhang Y., Yan H., Song X. (2019). Graphene/Si Schottky Solar Cells: A Review of Recent Advances and Prospects. RSC Adv..

[11] Song L., Yu X., Yang D. (2019). A Review on Graphene-Silicon Schottky Junction Interface. J. Alloy. Compd..

[12] Shin D.H., Choi S.-H. (2018). Use of Graphene for Solar Cells. J. Korean Phys. Soc..

[13] Abdullah M.F., Hashim A.M. (2019). Review and Assessment of Photovoltaic Performance of Graphene/Si Heterojunction Solar Cells. J. Mater. Sci..

[14] Ju S., Liang B., Wang J.-Z., Shi Y., Li S.-L. (2018). Graphene/Silicon Schottky Solar Cells: Technical Strategies for Performance Optimization. Opt. Commun..

[15] Cui K., Maruyama S. (2019). Multifunctional Graphene and Carbon Nanotube Films for Planar Heterojunction Solar Cells. Prog. Energy Combust. Sci..

[16] Shin D.H., Kwak G.Y., Kim J.M., Jang C.W., Choi S.-H., Kim K.J. (2019). Remarkable Enhancement of Stability in High-Efficiency Si-Quantum-Dot Heterojunction Solar Cells by Employing Bis(Trifluoromethanesulfonyl)-Amide as a Dopant for Graphene Transparent Conductive Electrodes. J. Alloy. Compd..

[17] Li X., Zhu H., Wang K., Cao A., Wei J., Li C., Jia Y., Li Z., Li X., Wu D. (2010). Graphene-On-Silicon Schottky Junction Solar Cells. Adv. Mater..

[18] Wirth-Lima A.J., Alves-Sousa P.P., Bezerra-Fraga W. (2020). N-Graphene/p-Silicon-Based Schottky Junction Solar Cell, with Very High Power Conversion Efficiency. SN Appl. Sci..

[19] Badhulika S., Terse-Thakoor T., Villarreal C., Mulchandani A. (2015). Graphene Hybrids: Synthesis Strategies and Applications in Sensors and Sensitized Solar Cells. Front. Chem..

[20] Haigh S.J., Gholinia A., Jalil R., Romani S., Britnell L., Elias D.C., Novoselov K.S., Ponomarenko L.A., Geim A.K., Gorbachev R. (2012). Cross-Sectional Imaging of Individual Layers and Buried Interfaces of Graphene-Based Heterostructures and Superlattices. Nat. Mater..

[21] Liang X., Sperling B.A., Calizo I., Cheng G., Hacker C.A., Zhang Q., Obeng Y., Yan K., Peng H., Li Q. (2011). Toward Clean and Crackless Transfer of Graphene. ACS Nano.

[22] Ihm K., Lim J.T., Lee K.-J., Kwon J.W., Kang T.-H., Chung S., Bae S., Kim J.H., Hong B.H., Yeom G.Y. (2010). Number of Graphene Layers as a Modulator of the Open-Circuit Voltage of Graphene-Based Solar Cell. Appl. Phys. Lett..

[23] Suhail A., Pan G., Jenkins D., Islam K. (2018). Improved Efficiency of Graphene/Si Schottky Junction Solar Cell Based on Back Contact Structure and DUV Treatment. Carbon.

[24] Li Y.F., Yang W., Tu Z.Q., Liu Z.C., Yang F., Zhang L.Q., Hatakeyama R. (2014). Schottky Junction Solar Cells Based on Graphene with Different Numbers of Layers. Appl. Phys. Lett..

[25] Li X., Xie D., Park H., Zeng T.H., Wang K., Wei J., Zhong M., Wu D., Kong J., Zhu H. (2013). Anomalous Behaviors of Graphene Transparent Conductors in Graphene-Silicon Heterojunction Solar Cells. Adv. Energy Mater..

[26] Das S., Pandey D., Thomas J., Roy T. (2019). The Role of Graphene and Other 2D Mater. in Solar Photovoltaics. Adv. Mater..

[27] Shin D.H., Kim J.H., Jung D.H., Choi S.-H. (2019). Graphene-Nanomesh Transparent Conductive Electrode/Porous-Si Schottky-Junction Solar Cells. J. Alloy. Compd..

[28] Lin Y.-K., Hong Y.-T., Shyue J.-J., Hsueh C.-H. (2019). Construction of Schottky Junction Solar Cell Using Silicon Nanowires and Multi-Layered Graphene. Superlattices Microstruct..

[29] Chugh S., Mehta R., Lu N., Dios F.D., Kim M.J., Chen Z. (2015). Comparison of Graphene Growth on Arbitrary Non-Catalytic Substrates Using Low-Temperature PECVD. Carbon.

[30] Jiao T., Liu J., Wei D., Feng Y., Song X., Shi H., Jia S., Sun W., Du C. (2015). Composite Transparent Electrode of Graphene Nanowalls and Silver Nanowires on Micropyramidal Si for High-Efficiency Schottky Junction Solar Cells. ACS Appl. Mater. Interfaces.

[31] Liu J., Sun W., Wei D., Song X., Jiao T., He S., Zhang W., Du C. (2015). Direct Growth of Graphene Nanowalls on the Crystalline Silicon for Solar Cells. Appl. Phys. Lett..

[32] Rehman M.A., Akhtar I., Choi W., Akbar K., Farooq A., Hussain S., Shehzad M.A., Chun S.-H., Jung J., Seo Y. (2018). Influence of an Al_2_O_3_ Interlayer in a Directly Grown Graphene-Silicon Schottky Junction Solar Cell. Carbon.

[33] Rehman M.A., Roy S.B., Akhtar I., Bhopal M.F., Choi W., Nazir G., Khan M.F., Kumar S., Eom J., Chun S.-H. (2019). Thickness-Dependent Efficiency of Directly Grown Graphene Based Solar Cells. Carbon.

[34] Rehman M.A., Roy S.B., Gwak D., Akhtar I., Nasir N., Kumar S., Khan M.F., Heo K., Chun S.-H., Seo Y. (2020). Solar Cell Based on Vertical Graphene Nano Hills Directly Grown on Silicon. Carbon.

[35] Bhopal M.F., von Lee D., Lee S.H., Lee A.R., Kim H.J., Lee S.H. (2019). Selective Nickel/Silver Front Metallization for Graphene/Silicon Solar Cells. Mater. Lett..

[36] Meng J.-H., Liu X., Zhang X.-W., Zhang Y., Wang H.-L., Yin Z.-G., Zhang Y.-Z., Liu H., You J.-B., Yan H. (2016). Interface Engineering for Highly Efficient Graphene-on-Silicon Schottky Junction Solar Cells by Introducing a Hexagonal Boron Nitride Interlayer. Nano Energy.

[37] Zhou Q., Liu X., Zhang E., Luo S., Shen J., Wang Y., Wei D. (2017). The Controlled Growth of Graphene Nanowalls on Si for Schottky Photodetector. AIP Adv..

[38] Jiao T., Wei D., Song X., Sun T., Yang J., Yu L., Feng Y., Sun W., Wei W., Shi H. (2016). High-Efficiency, Stable and Non-Chemically Doped Graphene–Si Solar Cells through Interface Engineering and PMMA Antireflection. RSC Adv..

[39] Gnisci A., Faggio G., Lancellotti L., Messina G., Carotenuto R., Bobeico E., Delli Veneri P., Capasso A., Dikonimos T., Lisi N. (2019). The Role of Graphene-Based Derivative as Interfacial Layer in Graphene/N-Si Schottky Barrier Solar Cells. Phys. Status Solidi A.

[40] Zhang L., Huang F., Li S., He S., Yu M., Fu J., Yang Q., Huang R., Cheng Q. (2020). Interface Engineering for Graphene Nanowalls/Silicon Schottky Solar Cells Prepared by Polymer-Free Transfer Method. J. Appl. Phys..

[41] Chandramohan S., Janardhanam V., Seo T.H., Hong C.-H., Suh E.-K. (2019). Improved Photovoltaic Effect in Graphene/Silicon Solar Cell Using MoO_3_/Ag/MoO_3_ Multilayer Coating. Mater. Lett..

[42] Miao X., Tongay S., Petterson M.K., Berke K., Rinzler A.G., Appleton B.R., Hebard A.F. (2012). High Efficiency Graphene Solar Cells by Chemical Doping. Nano Lett..

[43] Kern W. (1990). The Evolution of Silicon Wafer Cleaning Technology. J. Electrochem. Soc..

[44] Pour-mohammadi Z., Amirmazlaghani M. (2019). Asymmetric Finger-Shape Metallization in Graphene-on-Si Solar Cells for Enhanced Carrier Trapping. Mater. Sci. Semicond. Process..

[45] Kalita G., Wakita K., Umeno M., Tanemura M. (2013). Fabrication and Characteristics of Solution-Processed Graphene Oxide-Silicon Heterojunction. Phys. Status Solidi Rapid Res. Lett..

[46] Behura S.K., Nayak S., Mukhopadhyay I., Jani O. (2014). Junction Characteristics of Chemically-Derived Graphene/p-Si Heterojunction Solar Cell. Carbon.

[47] Merlen A., Buijnsters J.G., Pardanaud C. (2017). A Guide to and Review of the Use of Multiwavelength Raman Spectroscopy for Characterizing Defective Aromatic Carbon Solids: From Graphene to Amorphous Carbons. Coatings.

[48] Childres I., Jauregui L.A., Tian J., Chen Y.P. (2011). Effect of Oxygen Plasma Etching on Graphene Studied Using Raman Spectroscopy and Electronic Transport Measurements. New J. Phys..

[49] Hwang J.-S., Lin Y.-H., Hwang J.-Y., Chang R., Chattopadhyay S., Chen C.-J., Chen P., Chiang H.-P., Tsai T.-R., Chen L.-C. (2013). Imaging Layer Number and Stacking Order through Formulating Raman Fingerprints Obtained from Hexagonal Single Crystals of Few Layer Graphene. Nanotechnology.

[50] Mallet-Ladeira P., Puech P., Toulouse C., Cazayous M., Ratel-Ramond N., Weisbecker P., Vignoles G.L., Monthioux M. (2014). A Raman Study to Obtain Crystallite Size of Carbon Materials: A Better Alternative to the Tuinstra–Koenig Law. Carbon.

[51] Casiraghi C., Pisana S., Novoselov K.S., Geim A.K., Ferrari A.C. (2007). Raman Fingerprint of Charged Impurities in Graphene. Appl. Phys. Lett..

[52] Vinchon P., Glad X., Robert-Bigras G., Martel R., Sarkissian A., Stafford L. (2019). A Combination of Plasma Diagnostics and Raman Spectroscopy to Examine Plasma-Graphene Interactions in Low-Pressure Argon Radiofrequency Plasmas. J. Appl. Phys..

[53] Ferrari A.C., Meyer J.C., Scardaci V., Casiraghi C., Lazzeri M., Mauri F., Piscanec S., Jiang D., Novoselov K.S., Roth S. (2006). Raman Spectrum of Graphene and Graphene Layers. Phys. Rev. Lett..

[54] Eckmann A., Felten A., Mishchenko A., Britnell L., Krupke R., Novoselov K.S., Casiraghi C. (2012). Probing the Nature of Defects in Graphene by Raman Spectroscopy. Nano Lett..

[55] Khan A., Islam S.M., Ahmed S., Kumar R.R., Habib M.R., Huang K., Hu M., Yu X., Yang D. (2018). Direct CVD Growth of Graphene on Technologically Important Dielectric and Semiconducting Substrates. Adv. Sci..

[56] Stubrov Y., Nikolenko A., Strelchuk V., Nedilko S., Chornii V. (2017). Structural Modification of Single-Layer Graphene Under Laser Irradiation Featured by Micro-Raman Spectroscopy. Nanoscale Res. Lett..

[57] Nemes-Incze P., Osváth Z., Kamarás K., Biró L.P. (2008). Anomalies in Thickness Measurements of Graphene and Few Layer Graphite Crystals by Tapping Mode Atomic Force Microscopy. Carbon N Y.

[58] Yao Y., Ren L., Gao S., Li S. (2017). Histogram Method for Reliable Thickness Measurements of Graphene Films Using Atomic Force Microscopy (AFM). J. Mater. Sci. Technol..

[59] Kim S., Ryu S. (2016). Thickness-Dependent Native Strain in Graphene Membranes Visualized by Raman Spectroscopy. Carbon.

[60] Lee J.E., Ahn G., Shim J., Lee Y.S., Ryu S. (2012). Optical Separation of Mechanical Strain from Charge Doping in Graphene. Nat. Commun..

[61] Gayathri S., Jayabal P., Kottaisamy M., Ramakrishnan V. (2014). Synthesis of Few Layer Graphene by Direct Exfoliation of Graphite and a Raman Spectroscopic Study. AIP Adv..

[62] Tai L., Zhu D., Liu X., Yang T., Wang L., Wang R., Jiang S., Chen Z., Xu Z., Li X. (2018). Direct Growth of Graphene on Silicon by Metal-Free Chemical Vapor Deposition. Nano-Micro Lett..

[63] Neumann C., Reichardt S., Venezuela P., Drögeler M., Banszerus L., Schmitz M., Watanabe K., Taniguchi T., Mauri F., Beschoten B. (2015). Raman Spectroscopy as Probe of Nanometre-Scale Strain Variations in Graphene. Nat. Commun..

[64] Tang B., Guoxin H., Gao H. (2010). Raman Spectroscopic Characterization of Graphene. Appl. Spectrosc. Rev..

[65] Moon J.-Y., Kim M., Kim S.-I., Xu S., Choi J.-H., Whang D., Watanabe K., Taniguchi T., Park D.S., Seo J. (2020). Layer-Engineered Large-Area Exfoliation of Graphene. Sci. Adv..

[66] Wu J.-B., Lin M.-L., Cong X., Liu H.-N., Tan P.-H. (2018). Raman Spectroscopy of Graphene-Based Materials and Its Applications in Related Devices. Chem. Soc. Rev..

[67] Ribeiro-Soares J., Oliveros M.E., Garin C., David M.V., Martins L.G.P., Almeida C.A., Martins-Ferreira E.H., Takai K., Enoki T., Magalhães-Paniago R. (2015). Structural Analysis of Polycrystalline Graphene Systems by Raman Spectroscopy. Carbon.

[68] Pillet G., Sapelkin A., Bacsa W., Monthioux M., Puech P. (2019). Size-controlled Graphene-based Materials Prepared by Annealing of Pitch-based Cokes: G Band Phonon Line Broadening Effects Due to High Pressure, Crystallite Size, and Merging with D′ Band. J. Raman Spectrosc..

[69] Das A., Pisana S., Chakraborty B., Piscanec S., Saha S.K., Waghmare U.V., Novoselov K.S., Krishnamurthy H.R., Geim A.K., Ferrari A.C. (2008). Monitoring Dopants by Raman Scattering in an Electrochemically Top-Gated Graphene Transistor. Nat. Nanotechnol..

[70] Casiraghi C. (2009). Probing Disorder and Charged Impurities in Graphene by Raman Spectroscopy. Phys. Status Solidi Rapid Res. Lett..

[71] Fates R., Bouridah H., Raskin J.-P. (2019). Probing Carrier Concentration in Gated Single, Bi- and Tri-Layer CVD Graphene Using Raman Spectroscopy. Carbon.

[72] Lee U., Han Y., Lee S., Kim J.S., Lee Y.H., Kim U.J., Son H. (2020). Time Evolution Studies on Strain and Doping of Graphene Grown on a Copper Substrate Using Raman Spectroscopy. ACS Nano.

[73] Khalil H.M.W., Nam J.T., Kim K.S., Noh H. (2015). Controlled N-Doping in Chemical Vapour Deposition Grown Graphene by Antimony. J. Phys. D Appl. Phys..

[74] Bissett M.A., Tsuji M., Ago H. (2013). Mechanical Strain of Chemically Functionalized Chemical Vapor Deposition Grown Graphene. J. Phys. Chem. C.

[75] Gudaitis R., Lazauskas A., Jankauskas Š., Meškinis Š. (2020). Catalyst-Less and Transfer-Less Synthesis of Graphene on Si(100) Using Direct Microwave Plasma Enhanced Chemical Vapor Deposition and Protective Enclosures. Materials.

[76] Kiraly B., Jacobberger R.M., Mannix A.J., Campbell G.P., Bedzyk M.J., Arnold M.S., Hersam M.C., Guisinger N.P. (2015). Electronic and Mechanical Properties of Graphene–Germanium Interfaces Grown by Chemical Vapor Deposition. Nano Lett..

[77] Banszerus L., Janssen H., Otto M., Epping A., Taniguchi T., Watanabe K., Beschoten B., Neumaier D., Stampfer C. (2017). Identifying Suitable Substrates for High-Quality Graphene-Based Heterostructures. 2D Mater..

[78] Kang Y.-J., Kang J., Chang K.J. (2008). Electronic Structure of Graphene and Doping Effect on SiO_2_. Phys. Rev. B.

[79] Tung R.T. (2001). Recent Advances in Schottky Barrier Concepts. Mater. Sci. Eng. R Rep..

[80] Ali M.Y., Tao M. (2007). Effect of Sulfur Passivation of Silicon (100) on Schottky Barrier Height: Surface States versus Surface Dipole. J. Appl. Phys..

[81] Tao M., Udeshi D., Agarwal S., Maldonado E., Kirk W.P. (2004). Negative Schottky Barrier between Titanium and N-Type Si(001) for Low-Resistance Ohmic Contacts. Solid-State Electron..

[82] Martín I., Vetter M., Orpella A., Voz C., Puigdollers J., Alcubilla R., Kharchenko A.V., Roca i Cabarrocas P. (2004). Improvement of Crystalline Silicon Surface Passivation by Hydrogen Plasma Treatment. Appl. Phys. Lett..

[83] Soman A., Antony A. (2021). A Critical Study on Different Hydrogen Plasma Treatment Methods of A-Si: H/c-Si Interface for Enhanced Defect Passivation. Appl. Surf. Sci..

[84] Yamada T., Ohmi H., Okamoto K., Kakiuchi H., Yasutake K. (2012). Effects of Surface Temperature on High-Rate Etching of Silicon by Narrow-Gap Microwave Hydrogen Plasma. Jpn. J. Appl. Phys..

[85] Ishii M. (1994). Effects of Substrate Temperature and Bias Potential on Hydrogen Plasma Etching of Silicon. J. Vac. Sci. Technol. B Microelectron. Nanometer Struct..

[86] Schüttauf J.W.A., van der Werf C.H.M., van Sark W.G.J.H.M., Rath J.K., Schropp R.E.I. (2011). Comparison of Surface Passivation of Crystalline Silicon by A-Si:H with and without Atomic Hydrogen Treatment Using Hot-Wire Chemical Vapor Deposition. Thin Solid Film..

[87] Lavrov E.V., Weber J. (2001). Evolution of Hydrogen Platelets in Silicon Determined by Polarized Raman Spectroscopy. Phys. Rev. Lett..

[88] Zhu W., Low T., Perebeinos V., Bol A.A., Zhu Y., Yan H., Tersoff J., Avouris P. (2012). Structure and Electronic Transport in Graphene Wrinkles. Nano Lett..

[89] Zhong H., Liu Z., Shi L., Xu G., Fan Y., Huang Z., Wang J., Ren G., Xu K. (2014). Graphene in Ohmic Contact for Both N-GaN and p-GaN. Appl. Phys. Lett..

[90] Capasso A., Salamandra L., Faggio G., Dikonimos T., Buonocore F., Morandi V., Ortolani L., Lisi N. (2016). Chemical Vapor Deposited Graphene-Based Derivative as High-Performance Hole Transport Material for Organic Photovoltaics. ACS Appl. Mater. Interfaces.

[91] Yavuz S., Kuru C., Choi D., Kargar A., Jin S., Bandaru P.R. (2016). Graphene Oxide as a P-Dopant and an Anti-Reflection Coating Layer, in Graphene/Silicon Solar Cells. Nanoscale.

[92] Larsen L.J., Shearer C.J., Ellis A.V., Shapter J.G. (2016). Optimization and Doping of Reduced Graphene Oxide–Silicon Solar Cells. J. Phys. Chem. C.

[93] Adhikari S., Biswas C., Doan M.-H., Kim S.-T., Kulshreshtha C., Lee Y.H. (2019). Minimizing Trap Charge Density towards an Ideal Diode in Graphene–Silicon Schottky Solar Cell. ACS Appl. Mater. Interfaces.

[94] Song Y., Li X., Mackin C., Zhang X., Fang W., Palacios T., Zhu H., Kong J. (2015). Role of Interfacial Oxide in High-Efficiency Graphene–Silicon Schottky Barrier Solar Cells. Nano Lett..

[95] Zhong H., Xu K., Liu Z., Xu G., Shi L., Fan Y., Wang J., Ren G., Yang H. (2014). Charge Transport Mechanisms of Graphene/Semiconductor Schottky Barriers: A Theoretical and Experimental Study. J. Appl. Phys..

[96] Zhang X., Zhang L., Chan M. (2016). Doping Enhanced Barrier Lowering in Graphene-Silicon Junctions. Appl. Phys. Lett..

[97] Wittmann S., Aumer F., Wittmann D., Pindl S., Wagner S., Gahoi A., Reato E., Belete M., Kataria S., Lemme M.C. (2020). Dielectric Surface Charge Engineering for Electrostatic Doping of Graphene. ACS Appl. Electron. Mater..

[98] Shi Y., Dong X., Chen P., Wang J., Li L.-J. (2009). Effective Doping of Single-Layer Graphene from Underlying SiO_2_. Phys. Rev. B.

[99] Miwa R.H., Schmidt T.M., Scopel W.L., Fazzio A. (2011). Doping of Graphene Adsorbed on the A-SiO_2_ Surface. Appl. Phys. Lett..

[100] Samaddar S., Coraux J., Martin S.C., Grévin B., Courtois H., Winkelmann C.B. (2016). Equal Variations of the Fermi Level and Work Function in Graphene at the Nanoscale. Nanoscale.

[101] Leenaerts O., Partoens B., Peeters F.M., Volodin A., van Haesendonck C. (2017). The Work Function of Few-Layer Graphene. J. Phys. Condens. Matter.

[102] Ziegler D., Gava P., Güttinger J., Molitor F., Wirtz L., Lazzeri M., Saitta A.M., Stemmer A., Mauri F., Stampfer C. (2011). Variations in the Work Function of Doped Single- and Few-Layer Graphene Assessed by Kelvin Probe Force Microscopy and Density Functional Theory. Phys. Rev. B.

[103] Renault O., Pascon A.M., Rotella H., Kaja K., Mathieu C., Rault J.E., Blaise P., Poiroux T., Barrett N., Fonseca L.R.C. (2014). Charge Spill-out and Work Function of Few-Layer Graphene on SiC(0 0 0 1). J. Phys. D Appl. Phys..

[104] Akada K., Terasawa T., Imamura G., Obata S., Saiki K. (2014). Control of Work Function of Graphene by Plasma Assisted Nitrogen Doping. Appl. Phys. Lett..

[105] Sugaya T., Numakami O., Furue S., Komaki H., Amano T., Matsubara K., Okano Y., Niki S. (2011). Tunnel Current through a Miniband in InGaAs Quantum Dot Superlattice Solar Cells. Sol. Energy Mater. Sol. Cells.

[106] Wang Y., Wen Y., Sodabanlu H., Watanabe K., Sugiyama M., Nakano Y. (2012). A Superlattice Solar Cell with Enhanced Short-Circuit Current and Minimized Drop in Open-Circuit Voltage. IEEE J. Photovolt..

[107] Yin J., Liu L., Zang Y., Ying A., Hui W., Jiang S., Zhang C., Yang T., Chueh Y.-L., Li J. (2021). Engineered Tunneling Layer with Enhanced Impact Ionization for Detection Improvement in Graphene/Silicon Heterojunction Photodetectors. Light Sci. Appl..

[108] Xu J., Liu T., Hu H., Zhai Y., Chen K., Chen N., Li C., Zhang X. (2020). Design and Optimization of Tunneling Photodetectors Based on Graphene/Al2O3/Silicon Heterostructures. Nanophotonics.

[109] Zhou Y., Khan T.M., Shim J.W., Dindar A., Fuentes-Hernandez C., Kippelen B. (2014). All-Plastic Solar Cells with a High Photovoltaic Dynamic Range. J. Mater. Chem. A.

[110] Varghese A., Yakimov M., Tokranov V., Mitin V., Sablon K., Sergeev A., Oktyabrsky S. (2016). Complete Voltage Recovery in Quantum Dot Solar Cells Due to Suppression of Electron Capture. Nanoscale.

[111] Nishioka K., Sakitani N., Uraoka Y., Fuyuki T. (2007). Analysis of Multicrystalline Silicon Solar Cells by Modified 3-Diode Equivalent Circuit Model Taking Leakage Current through Periphery into Consideration. Sol. Energy Mater. Sol. Cells.

[112] Yang W., Luo Y., Guo P., Sun H., Yao Y. (2017). Leakage Current Induced by Energetic Disorder in Organic Bulk Heterojunction Solar Cells: Comprehending the Ultrahigh Loss of Open-Circuit Voltage at Low Temperatures. Phys. Rev. Appl..

[113] Tang Y., Bjuggren J.M., Fei Z., Andersson M.R., Heeney M., McNeill C.R. (2020). Origin of Open-Circuit Voltage Turnover in Organic Solar Cells at Low Temperature. Sol. RRL.

[114] Li Y., Yu M., Cheng Q. (2018). Improved Performance of Graphene/n-GaAs Heterojunction Solarcells by Introducing an Electron-Blocking/Hole-Transporting Layer. Mater. Res. Express.

[115] Armano A., Buscarino G., Cannas M., Gelardi F.M., Giannazzo F., Schilirò E., Agnello S. (2018). Monolayer Graphene Doping and Strain Dynamics Induced by Thermal Treatments in Controlled Atmosphere. Carbon.

[116] Bissett M.A., Izumida W., Saito R., Ago H. (2012). Effect of Domain Boundaries on the Raman Spectra of Mechanically Strained Graphene. ACS Nano.

[117] Frank O., Mohr M., Maultzsch J., Thomsen C., Riaz I., Jalil R., Novoselov K.S., Tsoukleri G., Parthenios J., Papagelis K. (2011). Raman 2D-Band Splitting in Graphene: Theory and Experiment. ACS Nano.

[118] Shiwakoti N., Bobby A., Asokan K., Antony B. (2018). Interface and Transport Properties of Gamma Irradiated Au/n-GaP Schottky Diode. Mater. Sci. Semicond. Processing.

[119] Becker J.A., Brattain W.H. (1934). The Thermionic Work Function and the Slope and Intercept of Richardson Plots. Phys. Rev..

[120] Lin T., Xie J., Ning S., Ma Z., Mu Y., Sun W., Yang S. (2022). Effect of Annealing Process Parameters on N-GaAs Ohmic Contacts. Microelectron. Eng..

[121] Lin T., Xie J., Ning S., Li Q., Li B. (2021). Study on the P-Type Ohmic Contact in GaAs-Based Laser Diode. Mater. Sci. Semicond. Processing.

[122] Latreche A. (2019). Combined Thermionic Emission and Tunneling Mechanisms for the Analysis of the Leakage Current for Ga_2_O_3_ Schottky Barrier Diodes. SN Appl. Sci..

[123] Arslan E., Çakmak H., Özbay E. (2012). Forward Tunneling Current in Pt/p-InGaN and Pt/n-InGaN Schottky Barriers in a Wide Temperature Range. Microelectron. Eng..

[124] An Y., Behnam A., Pop E., Ural A. (2013). Metal-Semiconductor-Metal Photodetectors Based on Graphene/p-Type Silicon Schottky Junctions. Appl. Phys. Lett..

[125] Tomer D., Rajput S., Hudy L.J., Li C.H., Li L. (2015). Carrier Transport in Reverse-Biased Graphene/Semiconductor Schottky Junctions. Appl. Phys. Lett..

