# Supplementary material for: The Graphene Structure’s Effects on the Current-Voltage and Photovoltaic Characteristics of Directly Synthesized Graphene/n-Si(100) Diodes
Source: Nanomaterials (Basel). 2022 May 11;12(10):1640. doi: 10.3390/nano12101640 (PMC9147930; doi:10.3390/nano12101640)
Supplement: Supplementary file 1 [file nanomaterials-12-01640-s001.zip › nanomaterials-1669946-supplementary.pdf]

nanomaterials

MDPI

Supplementary Materials

The Graphene Structure's Effects on the Current–Voltage and Photovoltaic Characteristics of Directly Synthesized Graphene/n-Si(100) Diodes

Šarūnas Jankauskas, Rimantas Gudaitis, Andrius Vasiliauskas, Asta Guobienė and Šarūnas Meškinis *

Institute of Materials Science, Kaunas University of Technology, K. Baršausko St. 59, LT-51423 Kaunas, Lithuania; sarunas.jankauskas@ktu.lt (Š.J.); rimantas.gudaitis@ktu.lt (R.G.); andrius.vasiliauskas@ktu.lt (A.V.); asta.guobiene@ktu.lt (A.G.)
* Correspondence: sarunas.meskinis@ktu.lt

Supplement S1. General information

Table S1. Summarized benchmark showing PCE values and PCE enhancement techniques of the CVD synthesized graphene/Si solar cells investigated by different research groups.

Ref.	Graphene synthesis method and type	Graphene doping	Interlayer	Light management technique	Power conversion efficiency, %
[1]	CVD on Cu foil and transfer onto n-type Si, planar graphene	bis(trifluoromethane sulfonyl)–amide	Si quantum dots	-	16.61%
[2]	CVD on Cu foil and transfer onto p-type Si, planar graphene	-	-	-	0.01%
[3]	CVD on Cu foil and transfer onto n-type Si, planar graphene	-	-	-	1.48%
[4]	CVD on Cu foil and transfer onto n-type Si, planar graphene	-	-	-	4.98%
[5]	CVD on Cu foil and transfer onto porous n-type Si, graphene nano-mesh	-	-	-	7.02%
[6]	PECVD on n-type Si micropyramids, graphene nanowalls	-	-	Surface texturing	6.6%
[7]	PECVD on n-type Si, graphene nanowalls	HNO_3	-	-	5.1%

[8]	PECVD on n-type Si, planar graphene	-	Al_2O_3	Surface texturing	8.4%
[9]	PECVD on n-type Si, planar graphene	-	-	Doped PMMA antireflecting coating	9.18%
[10]	PECVD on n-type Si, vertical graphene nanohills	PEDOT: PSS and inorganic acid HNO_3	Al_2O_3	Surface texturing	10.97%
[11]	PECVD on n-type Si, planar graphene	-	-	-	5.01%
[12]	Low-pressure CVD on Cu foil and transfer onto n-type Si	Au nanoparticles and HNO_3	h-BN	-	10.93%
[13]	PECVD on Cu foil and transfer onto n-type Si, GNWs	-	-	-	8.27%
[14]	Low pressure CVD on Cu foil and transfer onto n-type Si, planar grapehene	-	-	-	0.86%
[15]	CVD on Cu foil and transfer onto n-type Si(111), planar graphene	TFSA	Au/Cr contacts	-	1.9-3%

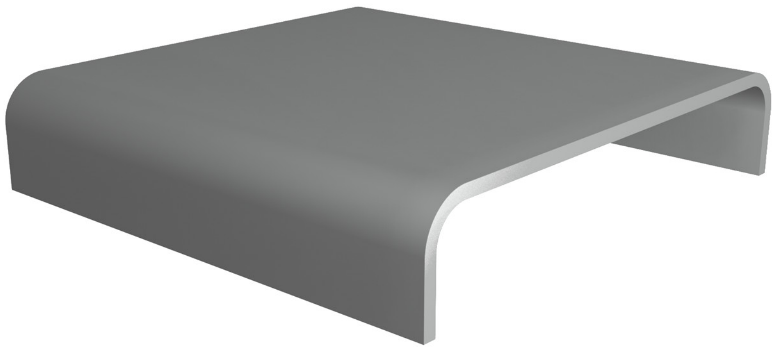

Figure S1. Schematic of an enclosure that was used during MW-PECVD process to prevent direct plasma effects.

Supplement S2. AFM measurements

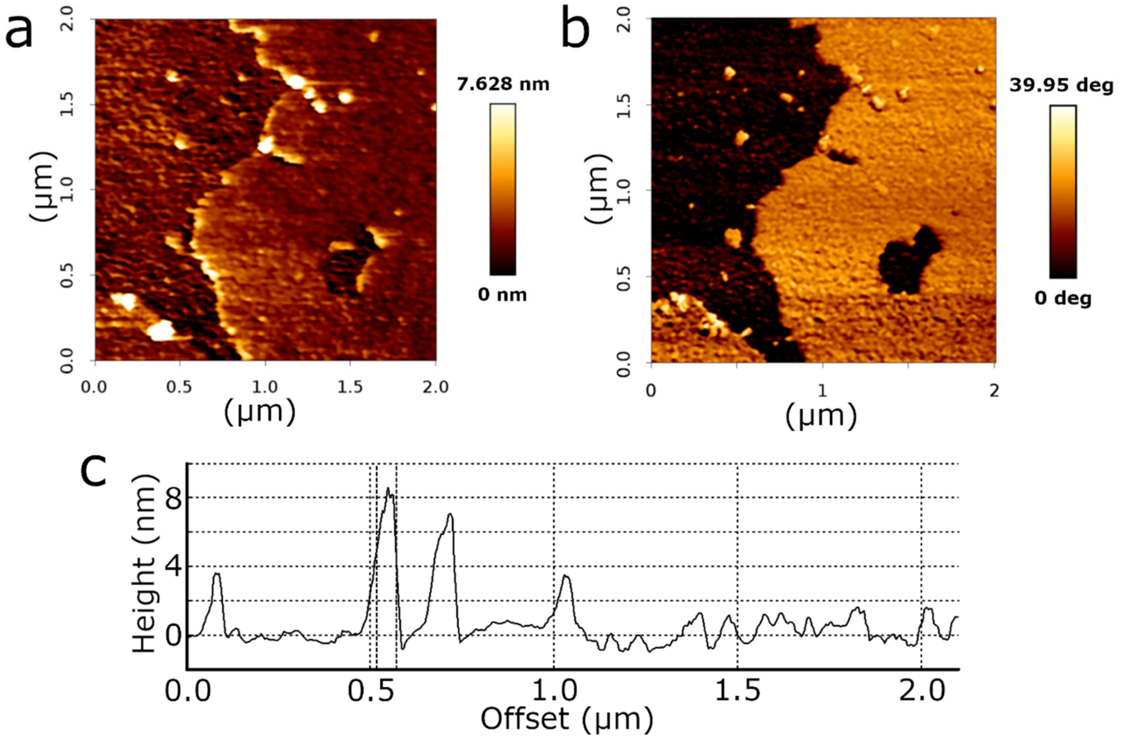

Figure S2. AFM image (**a**), AFM phase image (**b**) and height profile (**c**) of A1 sample.

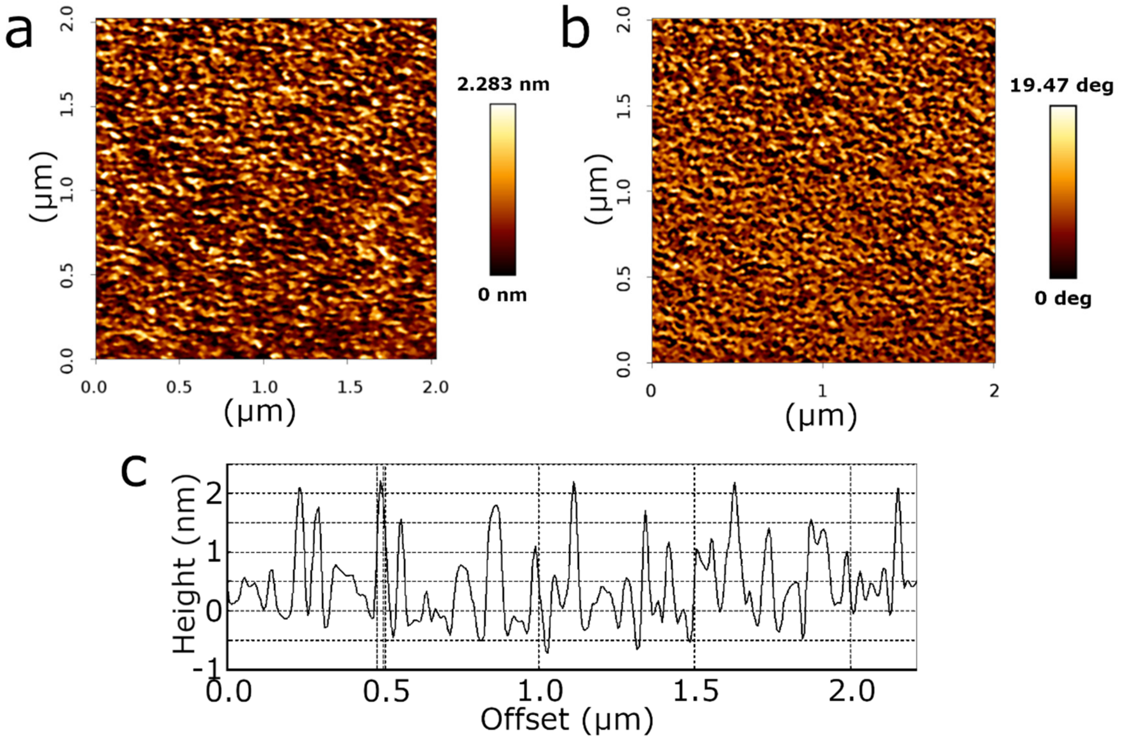

Figure S3. AFM image (**a**), AFM phase image (**b**) and height profile (**c**) of A7 sample.

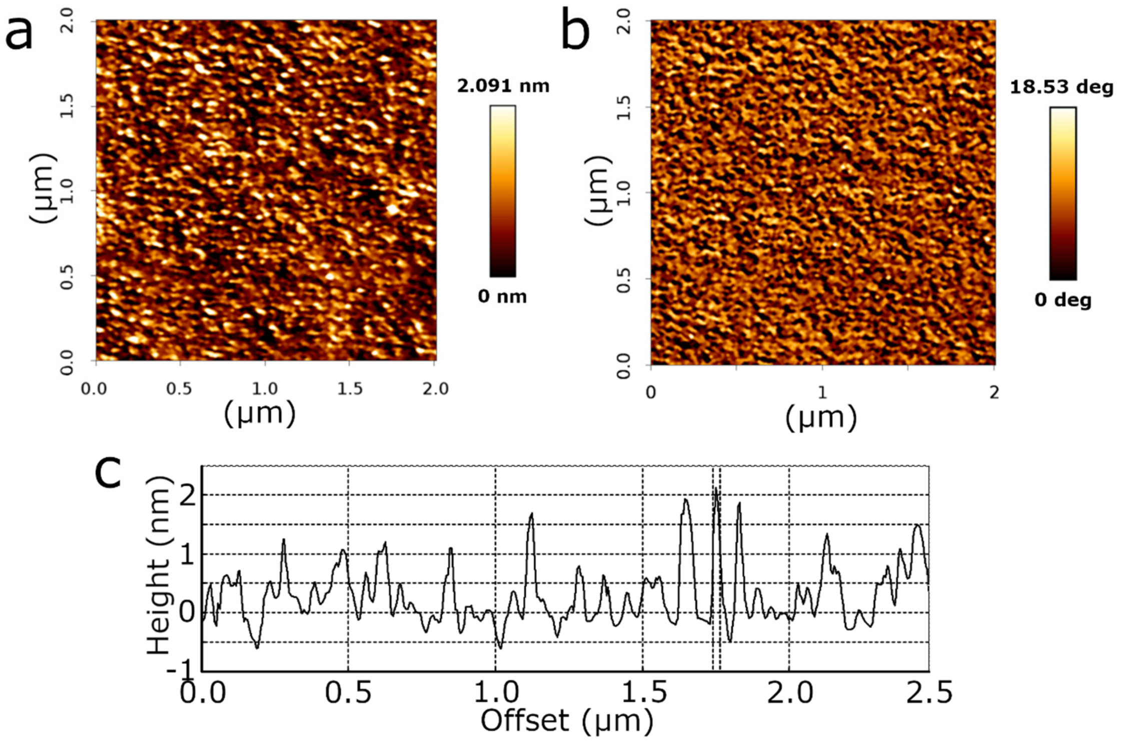

Figure S4. AFM image (**a**), AFM phase image (**b**) and height profile (**c**) of A8 sample.

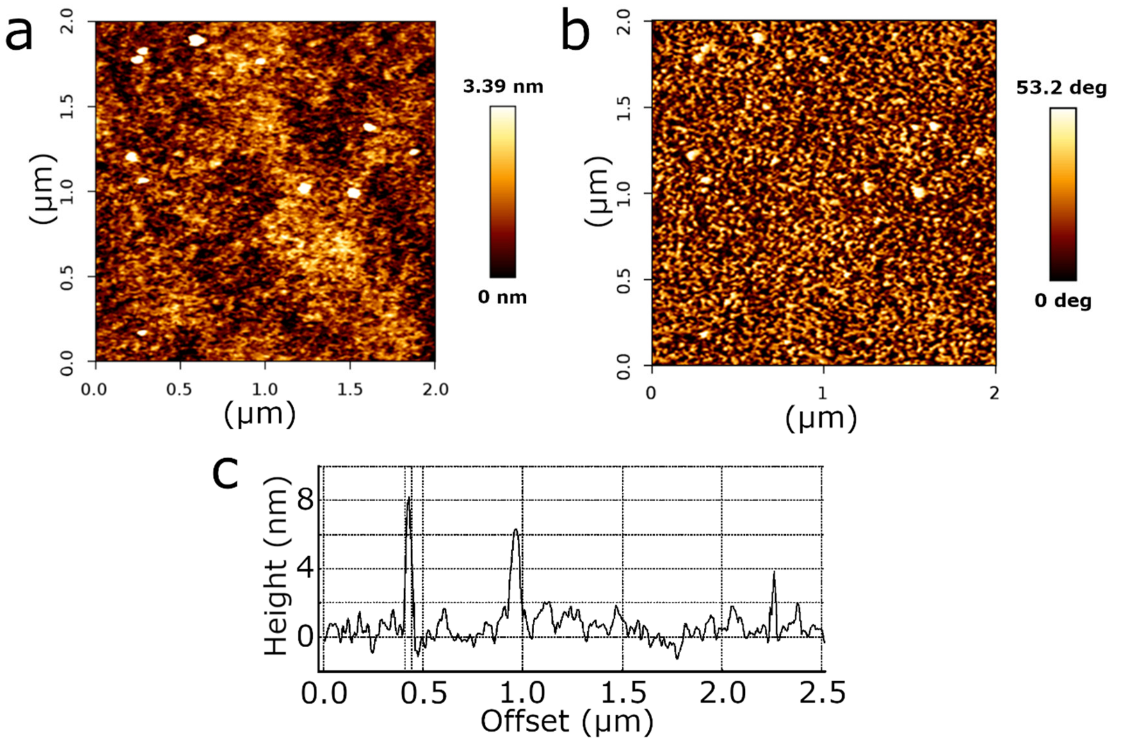

Figure S5. AFM image (**a**), AFM phase image (**b**) and height profile (**c**) of A11 sample.

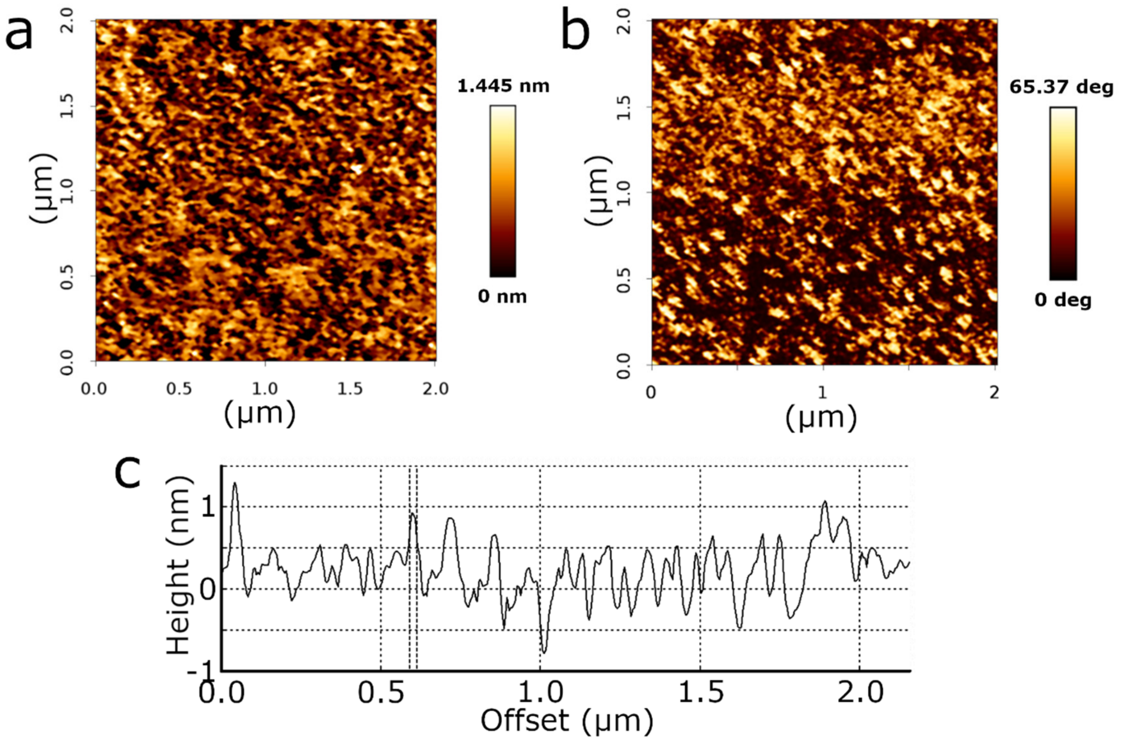

Figure S6. AFM image (**a**), AFM phase image (**b**) and height profile (**c**) of B1 sample.

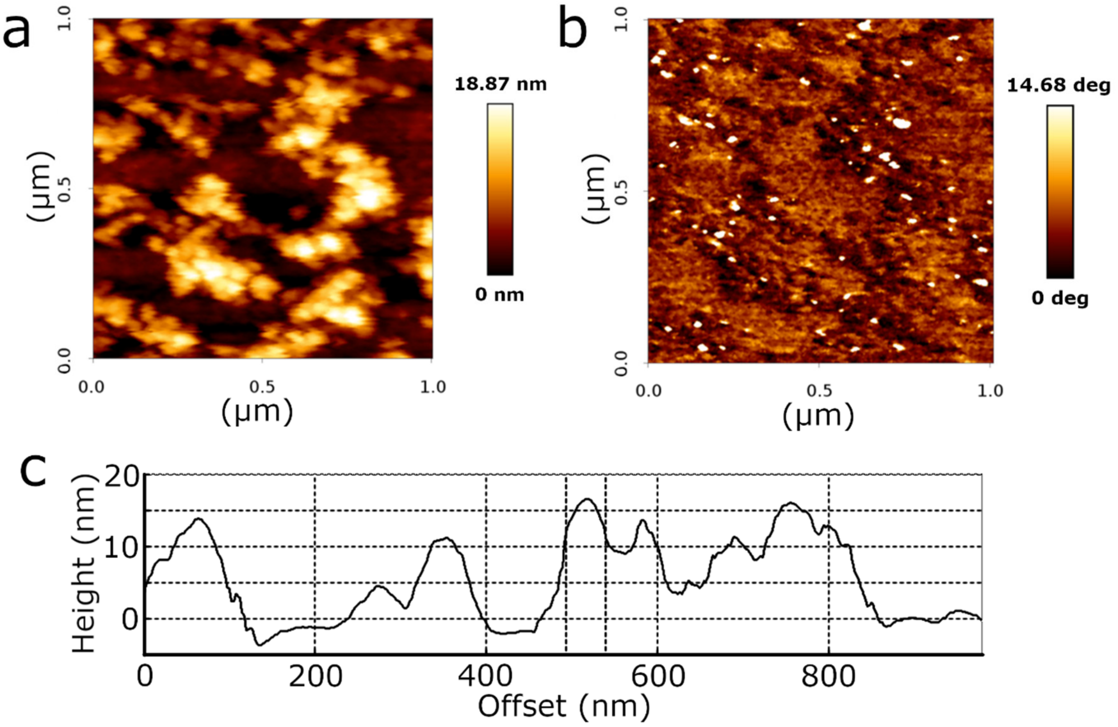

Figure S7. AFM image (**a**), AFM phase image (**b**) and height profile (**c**) of B2 sample.

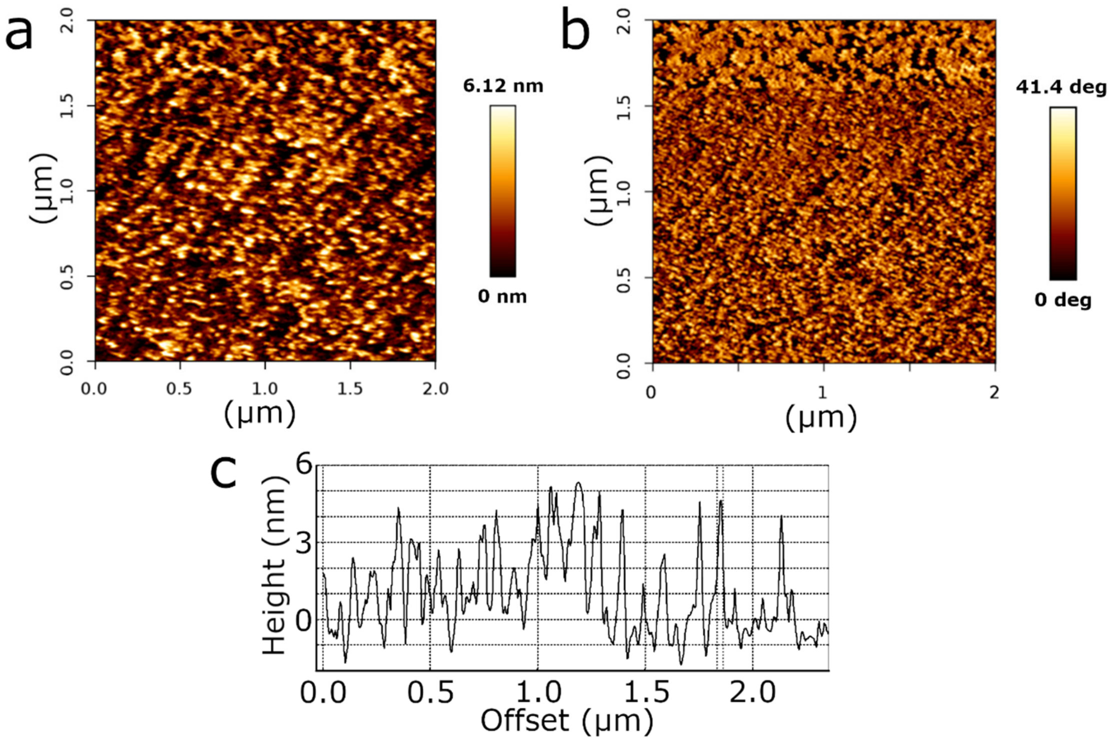

Figure S8. AFM image (**a**), AFM phase image (**b**) and height profile (**c**) of B3 sample.

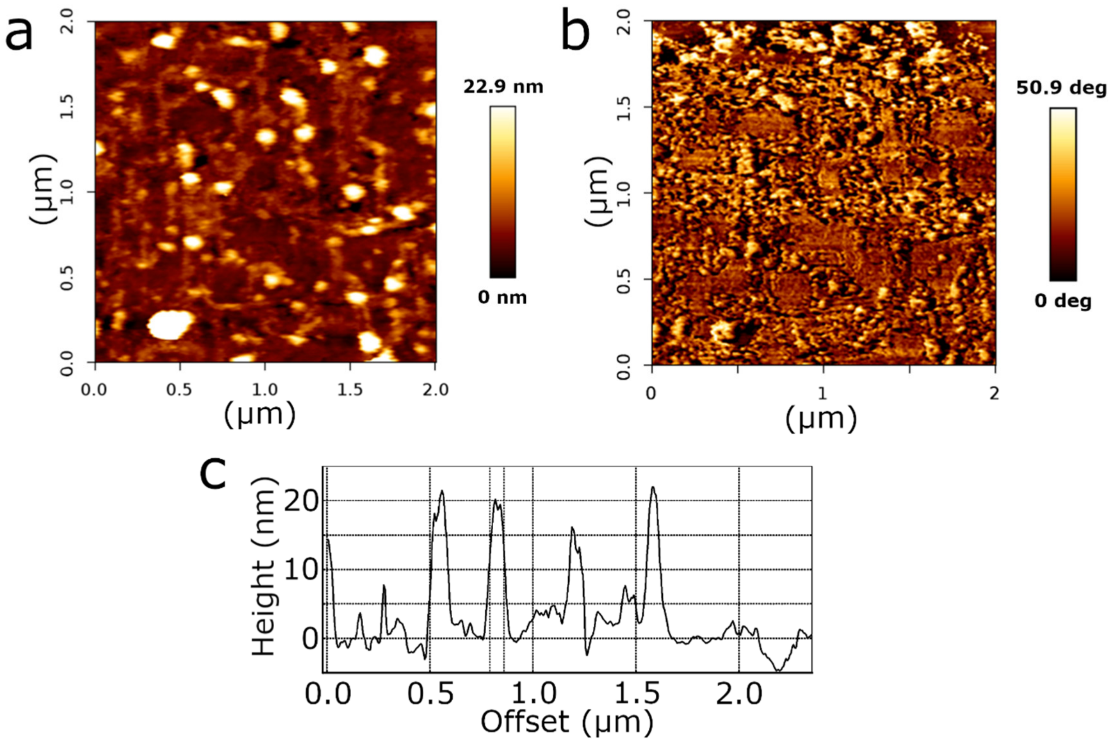

Figure S9. AFM image (**a**), AFM phase image (**b**) and height profile (**c**) of C2 sample.

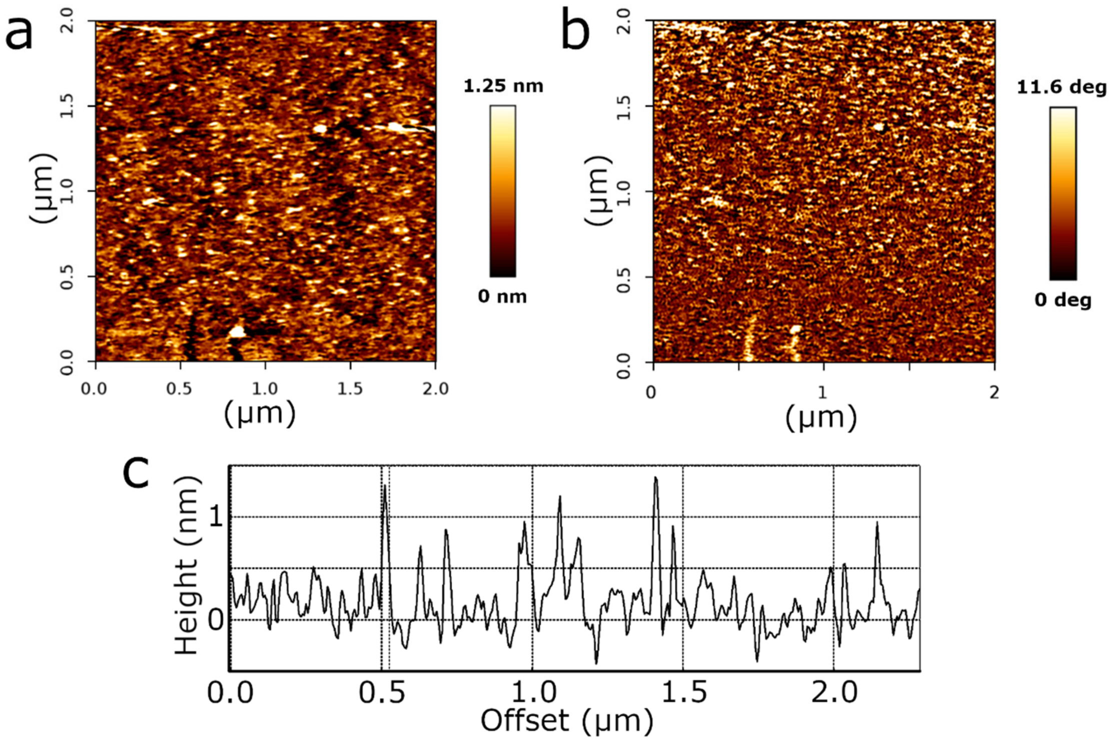

Figure S10. AFM image (**a**), AFM phase image (**b**) and height profile (**c**) of C3 sample.

Supplement S3. Structural and electrical properties

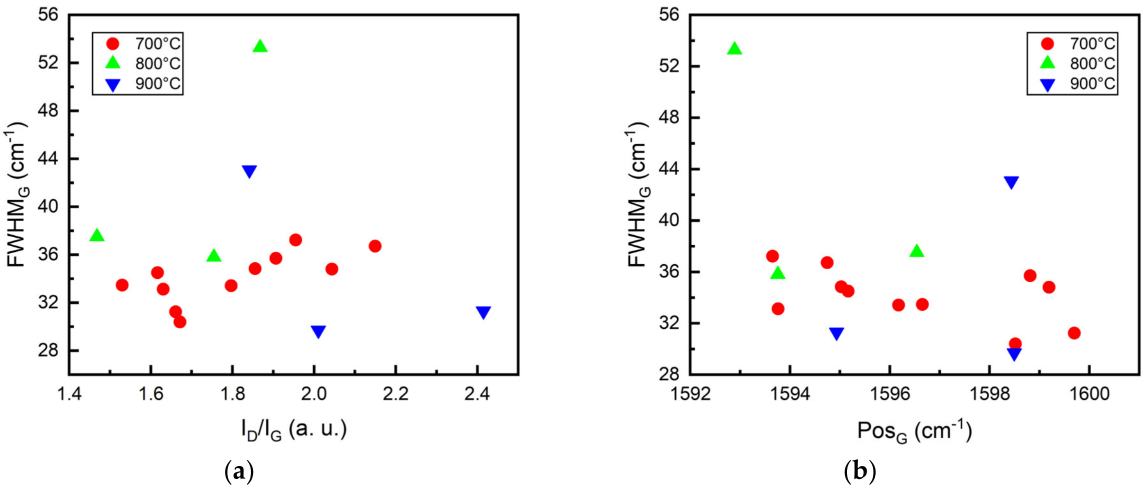

Figure S11. FWHM$_G$ vs. I_D/I_G (**a**) and FWHM$_G$ vs. Pos$_G$ (**b**) plots.

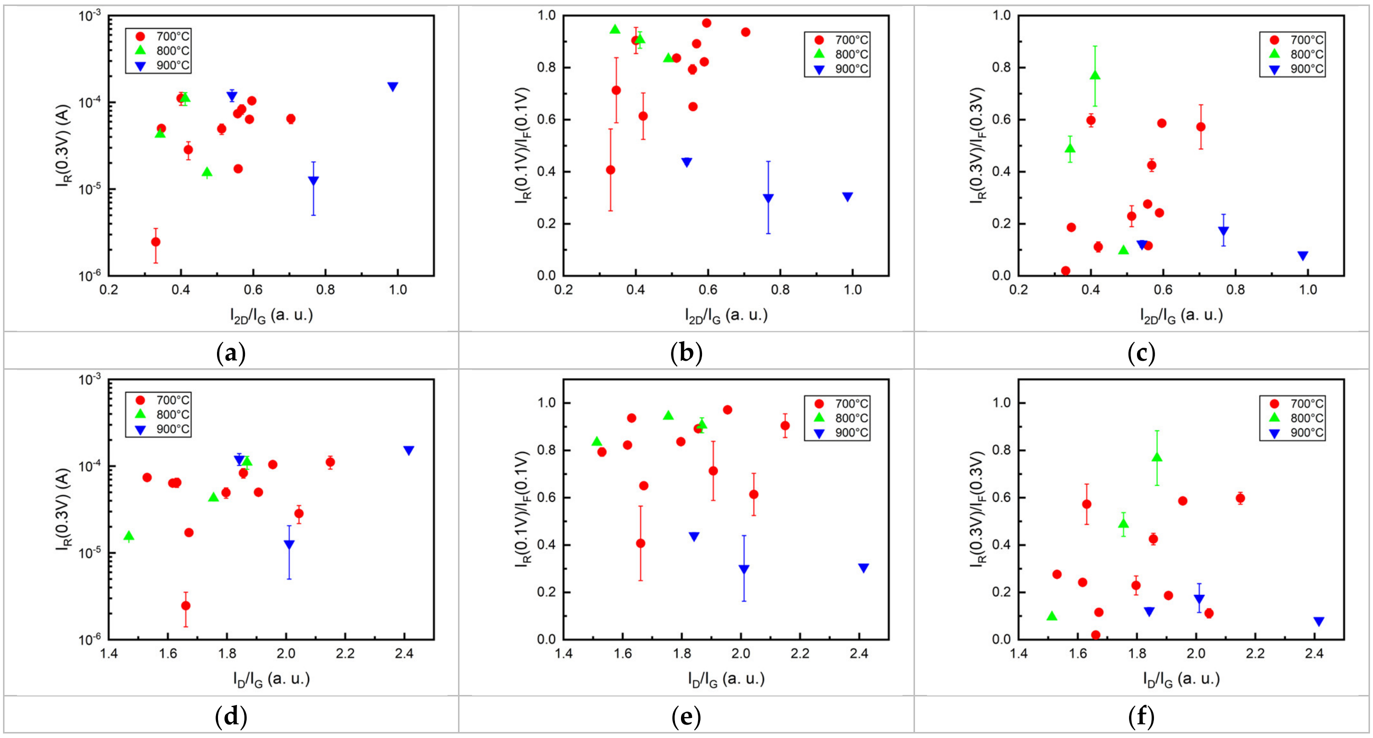

Figure S12. I-V characteristic parameters: (**a**,**d**) I_R(0.3 V); (**b**,**e**) I_R(0.1 V)/I_F(0.1 V); (**c**,**f**) I_R(0.3 V)/I_F(0.3 V); in relation with (**a**–**c**) I_{2D}/I_G; (**d**–**f**) I_D/I_G.

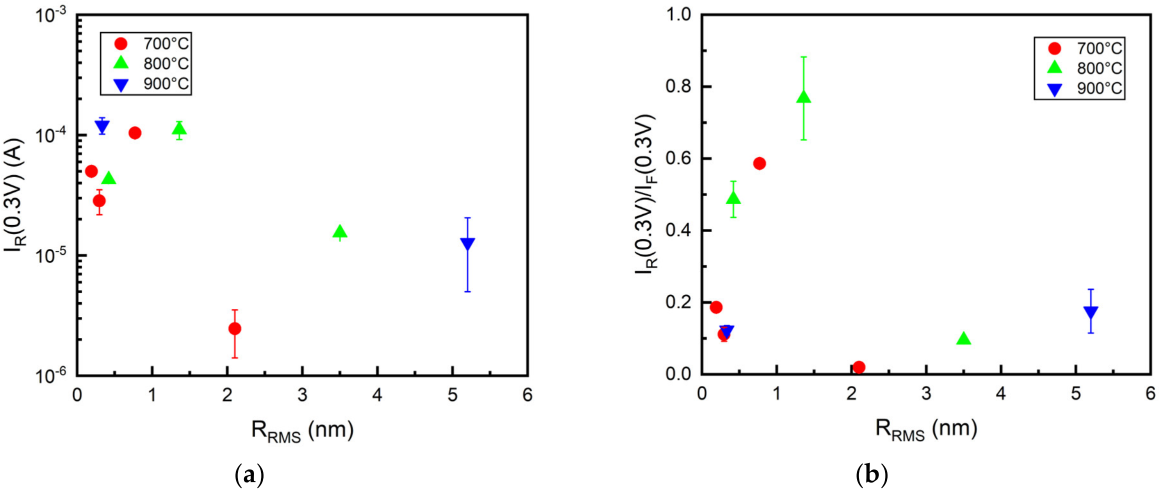

Figure S13. I-V characteristic parameter: (**a**) I_R(0.3 V); (**b**) I_R(0.3 V)/I_F(0.3 V) in relation with surface roughness.

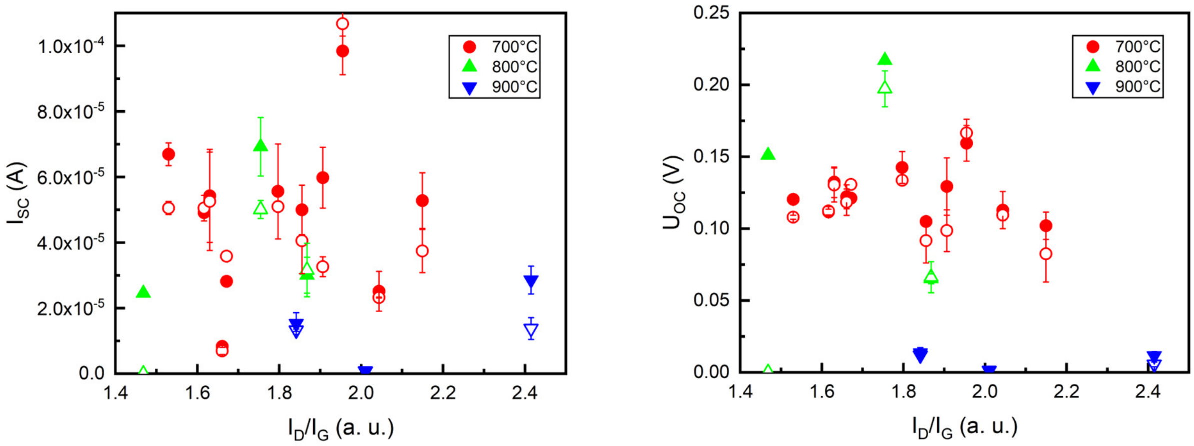

(**a**) (**b**)

Figure S14. I_{SC} vs. I_D/I_G (**a**) and U_{OC} vs. I_D/I_G (**b**) plot showing a difference between devices measured at 800 nm illumination (solid) and 406 nm illumination (hollow). In most cases at 406 nm illumination devices perform inferiorly than at 800 nm illumination.

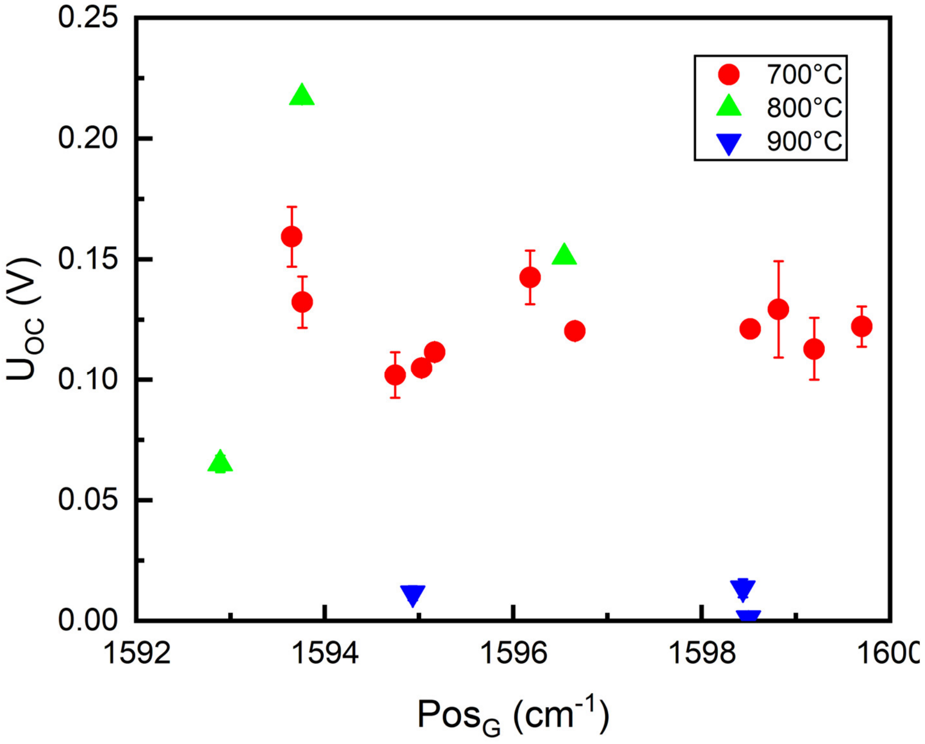

Figure S15. U_{OC} vs. Pos_G plot.

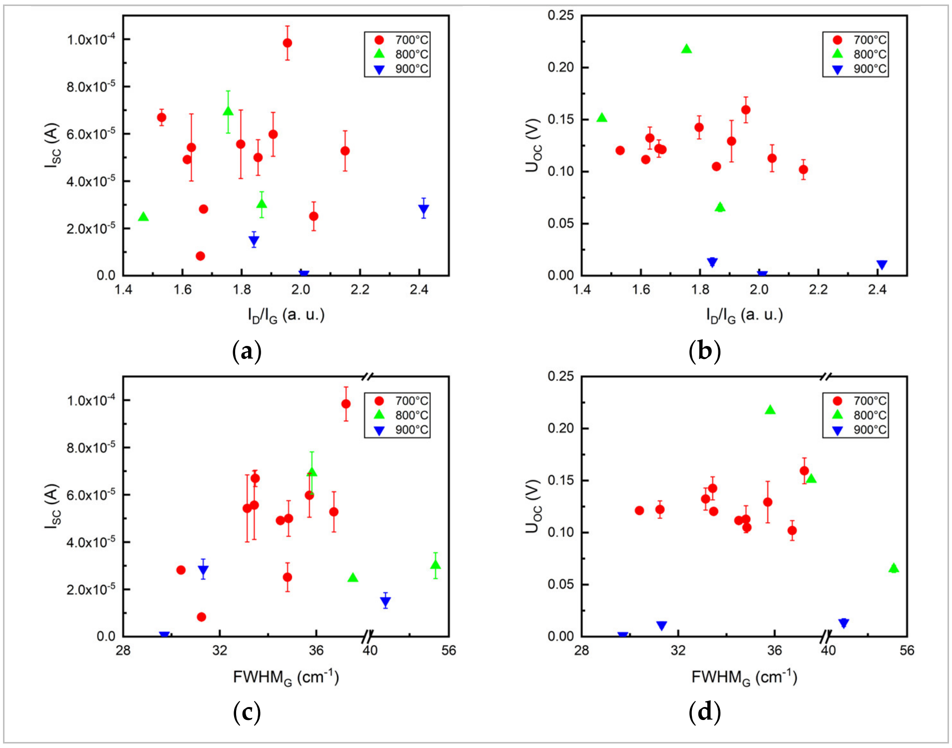

Figure S16. I_{SC} (**a**,**c**) and U_{OC} (**b**,**d**) relation with respect to I_D/I_G (**a**,**b**) and FWHM$_{2D}$ (**c**,**d**) under 800 nm illumination.

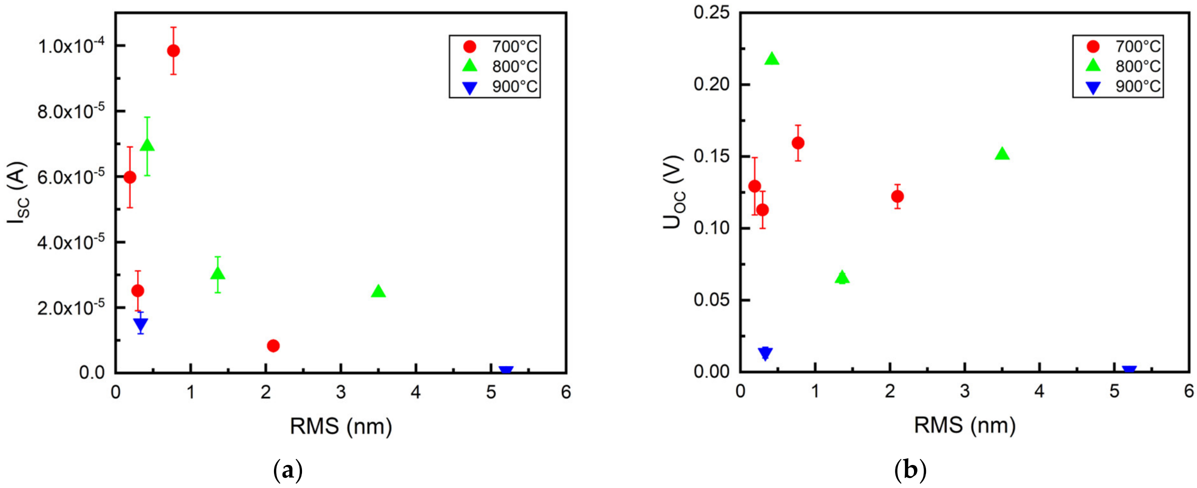

Figure S17. I_{SC} (**a**) and U_{OC} (**b**) and sample roughness relation under 800 nm illumination.

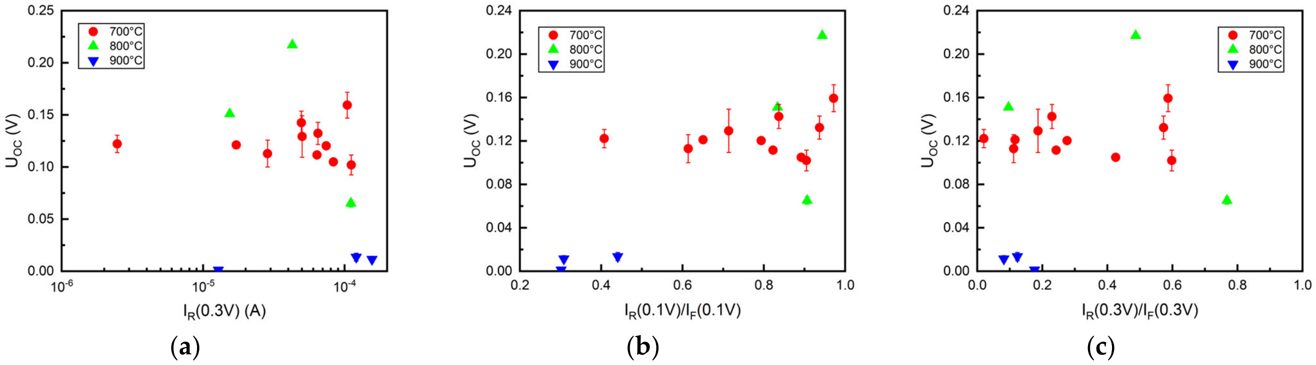

Figure S18. Diode I-V and U_{OC} (at 800 nm illumination) relation (**a**) U_{OC} vs. I_R(0.3 V) plot. U_{OC} correlation with diode's I-V curve shape: (**b**) reverse and forward current ratio at 0.1 V and (**c**) reverse and forward current ratio at 0.3 V.

Table S2. Probable doping and strain effects governing main graphene's Raman peak (G, 2D) positions and their FWHM.

Peak	p-type doping	n-type doping	Compressive stress	Tensile stress
Pos(G)	Upshift [16–21]	Upshift [16,17,21,22]	Upshift [18–20]	Upshift [23]
Pos(2D)	Upshift [16–21,24]	Downshift [16,17,21,22,24]	Upshift [18–20]	Upshift [23]
FWHM(G)	Narrowing [16,17,25,26]	Narrowing [16,17,22,25,26]	-	-
FWHM(2D)	Broadening [24]	Broadening [22,25]	Broadening [25,27,28]	Broadening [23,25,29]

Supplement S4. Diode operation in different thermal conditions, charge transport mechanisms

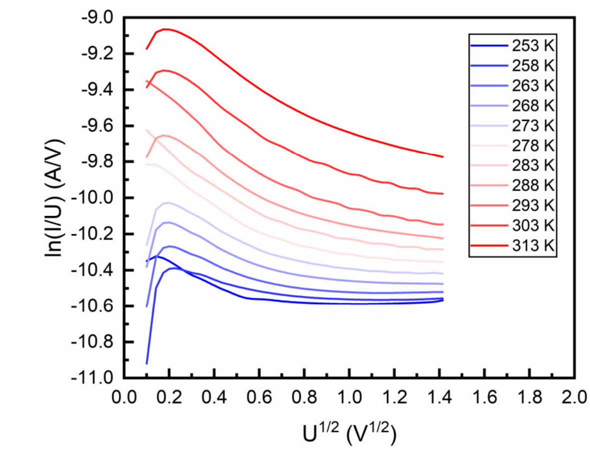

(**a**)

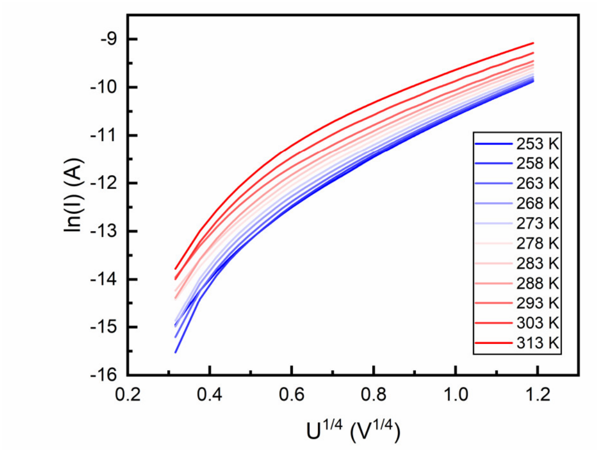

(**b**)

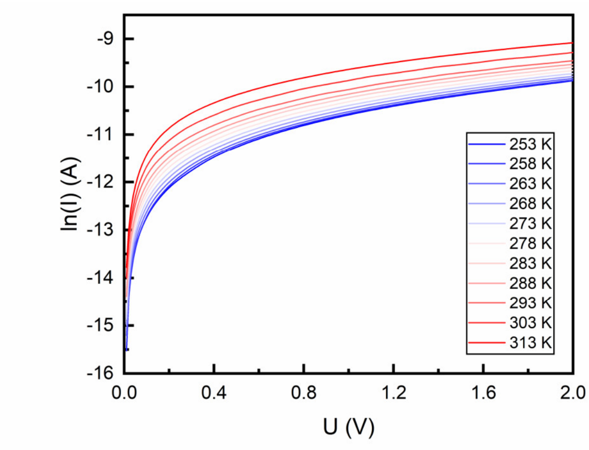

(**c**)

Figure S19. Different charge transport mechanisms estimated from typical fabricated diode I-V graphs under various thermal conditions: (**a**) Poole-Frenkel mechanism; (**b**) Image-Force induced charge transport; (**c**) Thermionic emission.

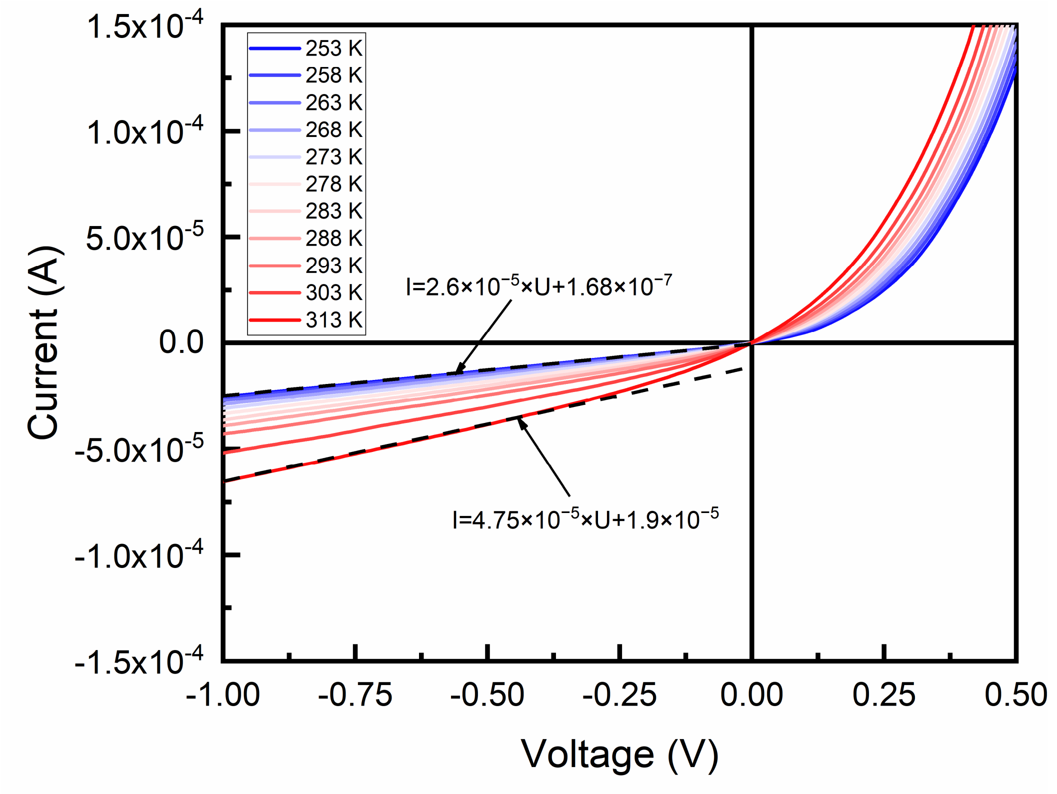

(**a**)

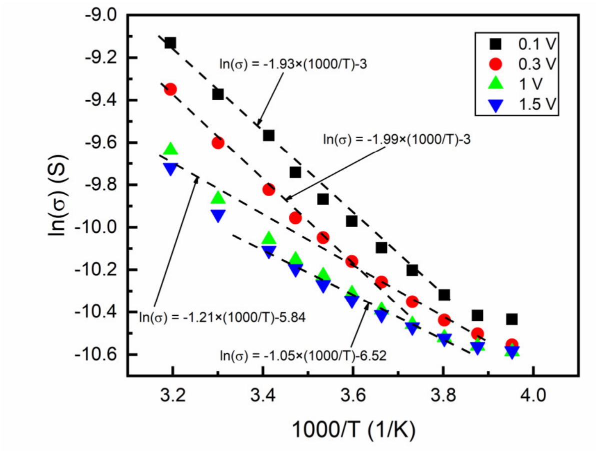

(**b**)

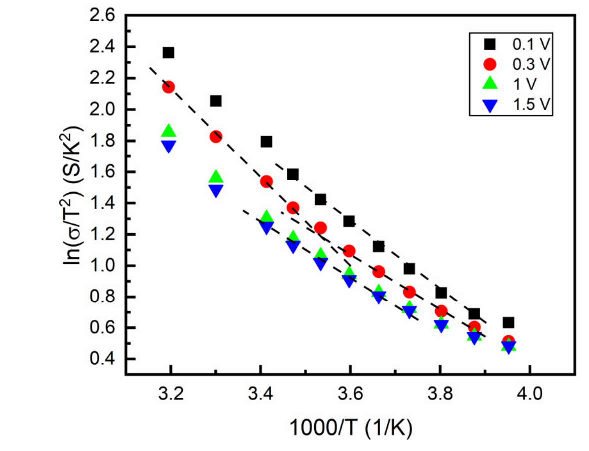

(**c**)

Figure S20. Diode operating regimes in terms of temperature: (**a**) typical I-V characteristics measured in the dark at different temperatures (253-313 K). Dashed black lines represent linear part of I-V curves. (**b**) The Arrhenius plot. (**c**) ln(σ/T^2) vs. 1000/T plot.

The typical current-voltage (I-V) characteristics measured in the dark at different temperatures are presented in Figure S20a. It can be seen that in all cases, the current increased with temperature. At the lowest temperatures, reverse I-V characteristics were linear in the all negative voltages range used. The linear range of the I-V characteristics decreased with measurement temperature. Reverse I-V characteristic measured at 40 °C temperature was nonlinear for up to -1 V voltage.

The Arrhenius plot ln(σ) vs. 1000/T was drawn (Figure S20b). The slope of the linear part of that plot is proportional to the charge carrier activation energy (E_a) and can be expressed as $\sigma = \sigma_0 e^{(-Ea/kT)}$ (see, e.g., [30]).

Thus, the presence of the two different charge carrier transport mechanisms, characterized by different activation energies, can be seen. Notably, one activation energy was

found at low measurement temperatures. It coincides with the range of the linear I-V characteristics. While at higher temperatures, in the ln(σ) vs. 1000/T plot, two different slope ranges can be seen, indicating the presence of the higher activation energy at lower reverse voltages.

One can see the linear ranges in the ln(σ/T^2) vs. 1000/T plot (Figure S20c). Such behavior is typical for thermionic emission current [31]. However, the ln(I) vs. U plot analysis revealed no linear range typical for thermionic emission. Thus, the superposition of the different charge transfer mechanisms should be supposed. Linear I-V characteristics can be related to the tunneling current flow (see, e.g. [32,33]). In such a way, competition between the thermionic emission and tunneling current can explain observed I-V characteristics and their dependence on measurement temperature. At low measurement temperature, the tunneling current dominates. The influence of the flow of the charge carriers over the potential barrier increase with measurement temperature. For I-V characteristics measured at higher temperatures, the current is dominated by the thermionic emission at low reverse biases, and at high biases, the tunneling current prevails. It should be mentioned that similar behavior was reported for various Schottky contacts [34,35] and graphene/silicon contacts [36,37].

Supplement S5. Hydrogen plasma pre-treatment effects

Table S3. Hydrogen plasma pre-treatment effects on Si(100) substrate surface.

Sample	Rq, nm	Phase Rq, °	Work function, eV
Reference Si	0.36	2.1	4.989
Si(100) plasma pre-treated at 700 °C temperature	0.35	1.4	4.993
Si(100) plasma pre-treated at 900 °C temperature	0.34	2.4	4.943

References

1. Shin, D.H.; Kwak, G.Y.; Kim, J.M.; Jang, C.W.; Choi, S.-H.; Kim, K.J. Remarkable Enhancement of Stability in High-Efficiency Si-Quantum-Dot Heterojunction Solar Cells by Employing Bis(Trifluoromethanesulfonyl)-Amide as a Dopant for Graphene Transparent Conductive Electrodes. *J. Alloys Compd.* **2019**, *773*, 913–918.
2. Ihm, K.; Lim, J.T.; Lee, K.-J.; Kwon, J.W.; Kang, T.-H.; Chung, S.; Bae, S.; Kim, J.H.; Hong, B.H.; Yeom, G.Y. Number of Graphene Layers as a Modulator of the Open-Circuit Voltage of Graphene-Based Solar Cell. *Appl. Phys. Lett.* **2010**, *97*, 032113.
3. Li, Y.F.; Yang, W.; Tu, Z.Q.; Liu, Z.C.; Yang, F.; Zhang, L.Q.; Hatakeyama, R. Schottky Junction Solar Cells Based on Graphene with Different Numbers of Layers. *Appl. Phys. Lett.* **2014**, *104*, 043903.
4. Li, X.; Xie, D.; Park, H.; Zeng, T.H.; Wang, K.; Wei, J.; Zhong, M.; Wu, D.; Kong, J.; Zhu, H. Anomalous Behaviors of Graphene Transparent Conductors in Graphene-Silicon Heterojunction Solar Cells. *Adv. Energy Mater.* **2013**, *3*, 1029–1034.
5. Shin, D.H.; Kim, J.H.; Jung, D.H.; Choi, S.-H. Graphene-Nanomesh Transparent Conductive Electrode/Porous-Si Schottky-Junction Solar Cells. *J. Alloys Compd.* **2019**, *803*, 958–963.
6. Jiao, T.; Liu, J.; Wei, D.; Feng, Y.; Song, X.; Shi, H.; Jia, S.; Sun, W.; Du, C. Composite Transparent Electrode of Graphene Nanowalls and Silver Nanowires on Micropyramidal Si for High-Efficiency Schottky Junction Solar Cells. *ACS Appl. Mater. Interfaces* **2015**, *7*, 20179–20183.
7. Liu, J.; Sun, W.; Wei, D.; Song, X.; Jiao, T.; He, S.; Zhang, W.; Du, C. Direct Growth of Graphene Nanowalls on the Crystalline Silicon for Solar Cells. *Appl. Phys. Lett.* **2015**, *106*, 043904.
8. Rehman, M.A.; Akhtar, I.; Choi, W.; Akbar, K.; Farooq, A.; Hussain, S.; Shehzad, M.A.; Chun, S.-H.; Jung, J.; Seo, Y. Influence of an Al2O3 Interlayer in a Directly Grown Graphene-Silicon Schottky Junction Solar Cell. *Carbon* **2018**, *132*, 157–164.
9. Rehman, M.A.; Roy, S.B.; Akhtar, I.; Bhopal, M.F.; Choi, W.; Nazir, G.; Khan, M.F.; Kumar, S.; Eom, J.; Chun, S.-H.; et al. Thickness-Dependent Efficiency of Directly Grown Graphene Based Solar Cells. *Carbon* **2019**, *148*, 187–195.
10. Rehman, M.A.; Roy, S.B.; Gwak, D.; Akhtar, I.; Nasir, N.; Kumar, S.; Khan, M.F.; Heo, K.; Chun, S.-H.; Seo, Y. Solar Cell Based on Vertical Graphene Nano Hills Directly Grown on Silicon. *Carbon* **2020**, *164*, 235–243.
11. Bhopal, M.F.; Lee, D. won; Lee, S.H.; Lee, A.R.; Kim, H.J.; Lee, S.H. Selective Nickel/Silver Front Metallization for Graphene/Silicon Solar Cells. *Mater. Lett.* **2019**, *234*, 237–240.
12. Meng, J.-H.; Liu, X.; Zhang, X.-W.; Zhang, Y.; Wang, H.-L.; Yin, Z.-G.; Zhang, Y.-Z.; Liu, H.; You, J.-B.; Yan, H. Interface Engineering for Highly Efficient Graphene-on-Silicon Schottky Junction Solar Cells by Introducing a Hexagonal Boron Nitride Interlayer. *Nano Energy* **2016**, *28*, 44–50.

13. Zhang, L.; Huang, F.; Li, S.; He, S.; Yu, M.; Fu, J.; Yang, Q.; Huang, R.; Cheng, Q. Interface Engineering for Graphene Nanowalls/Silicon Schottky Solar Cells Prepared by Polymer-Free Transfer Method. *J. Appl. Phys.* **2020**, *128*, 025301.
14. Chandramohan, S.; Janardhanam, V.; Seo, T.H.; Hong, C.-H.; Suh, E.-K. Improved Photovoltaic Effect in Graphene/Silicon Solar Cell Using MoO3/Ag/MoO3 Multilayer Coating. *Mater. Lett.* **2019**, *246*, 103–106.
15. Miao, X.; Tongay, S.; Petterson, M.K.; Berke, K.; Rinzler, A.G.; Appleton, B.R.; Hebard, A.F. High Efficiency Graphene Solar Cells by Chemical Doping. *Nano Lett.* **2012**, *12*, 2745–2750.
16. Das, A.; Pisana, S.; Chakraborty, B.; Piscanec, S.; Saha, S.K.; Waghmare, U. v.; Novoselov, K.S.; Krishnamurthy, H.R.; Geim, A.K.; Ferrari, A.C.; et al. Monitoring Dopants by Raman Scattering in an Electrochemically Top-Gated Graphene Transistor. *Nat. Nanotechnol.* **2008**, *3*, 210–215.
17. Casiraghi, C. Probing Disorder and Charged Impurities in Graphene by Raman Spectroscopy. *Phys. Status Solidi Rapid Res. Lett.* **2009**, *3*, 175–177.
18. Lee, U.; Han, Y.; Lee, S.; Kim, J.S.; Lee, Y.H.; Kim, U.J.; Son, H. Time Evolution Studies on Strain and Doping of Graphene Grown on a Copper Substrate Using Raman Spectroscopy. *ACS Nano* **2020**, *14*, 919–926.
19. Armano, A.; Buscarino, G.; Cannas, M.; Gelardi, F.M.; Giannazzo, F.; Schilirò, E.; Agnello, S. Monolayer Graphene Doping and Strain Dynamics Induced by Thermal Treatments in Controlled Atmosphere. *Carbon* **2018**, *127*, 270–279.
20. Kim, S.; Ryu, S. Thickness-Dependent Native Strain in Graphene Membranes Visualized by Raman Spectroscopy. *Carbon* **2016**, *100*, 283–290.
21. Lee, J.E.; Ahn, G.; Shim, J.; Lee, Y.S.; Ryu, S. Optical Separation of Mechanical Strain from Charge Doping in Graphene. *Nat. Commun.* **2012**, *3*, 1024.
22. Khalil, H.M.W.; Nam, J.T.; Kim, K.S.; Noh, H. Controlled N-Doping in Chemical Vapour Deposition Grown Graphene by Antimony. *J. Phys. D: Appl. Phys.* **2015**, *48*, 015307.
23. Moon, J.-Y.; Kim, M.; Kim, S.-I.; Xu, S.; Choi, J.-H.; Whang, D.; Watanabe, K.; Taniguchi, T.; Park, D.S.; Seo, J.; et al. Layer-Engineered Large-Area Exfoliation of Graphene. *Sci. Adv.* **2020**, *6*.
24. Tang, B.; Guoxin, H.; Gao, H. Raman Spectroscopic Characterization of Graphene. *Appl. Spectrosc. Rev.* **2010**, *45*, 369–407.
25. Neumann, C.; Reichardt, S.; Venezuela, P.; Drögeler, M.; Banszerus, L.; Schmitz, M.; Watanabe, K.; Taniguchi, T.; Mauri, F.; Beschoten, B.; et al. Raman Spectroscopy as Probe of Nanometre-Scale Strain Variations in Graphene. *Nat. Commun.* **2015**, *6*, 8429.
26. Fates, R.; Bouridah, H.; Raskin, J.-P. Probing Carrier Concentration in Gated Single, Bi- and Tri-Layer CVD Graphene Using Raman Spectroscopy. *Carbon* **2019**, *149*, 390–399.
27. Bissett, M.A.; Tsuji, M.; Ago, H. Mechanical Strain of Chemically Functionalized Chemical Vapor Deposition Grown Graphene. *J. Phys. Chem.* C **2013**, *117*, 3152–3159.
28. Bissett, M.A.; Izumida, W.; Saito, R.; Ago, H. Effect of Domain Boundaries on the Raman Spectra of Mechanically Strained Graphene. *ACS Nano* **2012**, *6*, 10229–10238.
29. Frank, O.; Mohr, M.; Maultzsch, J.; Thomsen, C.; Riaz, I.; Jalil, R.; Novoselov, K.S.; Tsoukleri, G.; Parthenios, J.; Papagelis, K.; et al. Raman 2D-Band Splitting in Graphene: Theory and Experiment. *ACS Nano* **2011**, *5*, 2231–2239.
30. Shiwakoti, N.; Bobby, A.; Asokan, K.; Antony, B. Interface and Transport Properties of Gamma Irradiated Au/n-GaP Schottky Diode. *Materials Science in Semiconductor Processing* **2018**, *74*, 1–6.
31. Becker, J.A.; Brattain, W.H. The Thermionic Work Function and the Slope and Intercept of Richardson Plots. *Physical Review* **1934**, *45*, 694–705.
32. Lin, T.; Xie, J.; Ning, S.; Ma, Z.; Mu, Y.; Sun, W.; Yang, S. Effect of Annealing Process Parameters on N-GaAs Ohmic Contacts. *Microelectronic Engineering* **2022**, *258*, 111772.
33. Lin, T.; Xie, J.; Ning, S.; Li, Q.; Li, B. Study on the P-Type Ohmic Contact in GaAs-Based Laser Diode. *Materials Science in Semiconductor Processing* **2021**, *124*, 105622.
34. Latreche, A. Combined Thermionic Emission and Tunneling Mechanisms for the Analysis of the Leakage Current for Ga2O3 Schottky Barrier Diodes. *SN Applied Sciences* **2019**, *1*, 188.
35. Arslan, E.; Çakmak, H.; Özbay, E. Forward Tunneling Current in Pt/p-InGaN and Pt/n-InGaN Schottky Barriers in a Wide Temperature Range. *Microelectronic Engineering* **2012**, *100*, 51–56.
36. An, Y.; Behnam, A.; Pop, E.; Ural, A. Metal-Semiconductor-Metal Photodetectors Based on Graphene/p-Type Silicon Schottky Junctions. *Applied Physics Letters* **2013**, *102*, 013110.
37. Tomer, D.; Rajput, S.; Hudy, L.J.; Li, C.H.; Li, L. Carrier Transport in Reverse-Biased Graphene/Semiconductor Schottky Junctions. *Applied Physics Letters* **2015**, *106*, 173510.
